# Improved normalization of lesioned brains via cohort‐specific templates

**DOI:** 10.1002/hbm.25474

**Published:** 2021-06-18

**Authors:** Ioannis Pappas, Henrik Hector, Kari Haws, Brian Curran, Andrew S. Kayser, Mark D'Esposito

**Affiliations:** ^1^ Department of Psychology Helen Wills Neuroscience Institute, University of California, Berkeley Berkeley California USA; ^2^ Department of Neurology VA Northern California Health Care System Martinez California USA; ^3^ Department of Neurology University of California, San Francisco San Francisco California USA; ^4^ Department of Psychology University of California, Berkeley Berkeley California USA

**Keywords:** algorithm, diffeomorphism, fMRI, lesion, normalization, stroke, template

## Abstract

In MRI studies, spatial normalization is required to infer results at the group level. In the presence of a brain lesion, such as in stroke patients, the normalization process can be affected by tissue loss, spatial deformations, signal intensity changes, and other stroke sequelae that introduce confounds into the group analysis results. Previously, most neuroimaging studies with lesioned brains have used normalization methods optimized for intact brains, raising potential concerns about the accuracy of the resulting transformations and, in turn, their reported group level results. In this study, we demonstrate the benefits of creating an intermediate, cohort‐specific template in conjunction with diffeomorphism‐based methods to normalize structural MRI images in stroke patients. We show that including this cohort‐specific template improves accuracy compared to standard methods for normalizing lesioned brains. Critically, this method reduces overall differences in normalization accuracy between stroke patients and healthy controls, and may improve the localization and connectivity of BOLD signal in functional neuroimaging data.

## Graphical abstract

Proper normalization is crucial for group neuroimaging studies. However, this process is often suboptimal in lesion studies. We propose that the normalizing images to a cohort‐specific template may improve normalization of lesion studies and increase the fidelity of the group level results.

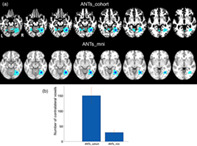

## 1 INTRODUCTION

Group studies of neuroimaging data depend on the ability to compare homologous brain regions across individuals. The most common approach for conducting group analyses in neuroimaging studies is to transform individuals' brain images to match a common template, typically the Montreal Neurological Institute (MNI‐152) template derived from 152 healthy individuals (Brett, Johnsrude, & Owen, 2002). This process of registering individual brains to a common space is referred to as normalization, in which mathematical transformations optimize the match between the brain image and the template (Friston et al., 1995).

Previously, different normalization techniques have been developed in the form of complex optimization problems. The primary goal of these techniques is to maximize similarity between the template and the image given an underlying objective function and its parameterization. Nonlinear algorithms have been an important advancement, allowing complicated transformations for matching local structures based on high parameterization of the warp (Ashburner & Friston, 1999). One of the most popular techniques is the unified segmentation implemented in the Statistical Parametric Mapping (SPM) software package (Ashburner & Friston, 2007). In part, this technique improves normalization over affine methods by segmenting the brain tissue based on prior probability distributions for white matter, gray matter and cerebrospinal fluid tissue maps in MNI‐152 space (Ashburner & Friston, 2007).

These methods include on the order of 10^3^ parameters when trying to maximize similarity between individual brains and the MNI‐152 space. However, Hellier and colleagues demonstrated that the number of degrees of freedom of the nonlinear transformation is directly related to normalization performance (Hellier et al., 2003), and that standard nonlinear algorithms failed to capture both idiosyncratic local deformations (e.g., for distinctive shapes of cortical sulci) and larger overall differences in brain structure. To better match the dissimilar topologies between individual and template images, more complex methods have emerged that include large deformations and millions of parameters in their objective functions. If these deformations are invertible, diffeomorphic and parameterized by a flow field, then the registration process can be described by a closed solution that can be used to maximize similarity between the brain and MNI images (Avants, Epstein, Grossman, & Gee, 2008). These approaches have been implemented in the Advanced Normalization Tools (ANTS) toolbox (Avants et al., 2011), which utilizes a powerful diffeomorphism‐based algorithm for normalization, namely the Symmetric Normalization algorithm (SyN) (Avants et al., 2008). The SyN algorithm maximizes similarity between the image and the template by finding the optimal diffeomorphism between them using specific optimization techniques, with additional assumptions about the symmetry of the diffeomorphism (Avants, Anderson, Grossman, & Gee, 2007). More recently, formal comparisons of the differential normalization accuracy of a number of normalization methods have been undertaken. In the most comprehensive study in the literature, researchers tested 14 nonlinear, deformation‐based normalization algorithms on a large cohort of healthy subjects, with SyN and another algorithm, ART, emerging as the most consistently accurate (Klein et al., 2009).

In contrast with the normalization of healthy subjects' brain images, the presence of lesions causes large structural deformations that can occur in a complicated, nonlinear fashion. This gross mismatch between a lesioned brain and the template significantly impacts normalization accuracy, and necessitates the amendment of previous normalization methods. Of all the approaches addressing this issue, cost‐function masking (i.e., registration by “masking” out the lesioned tissue) is the most prevalent (Brett, Leff, Rorden, & Ashburner, 2001). However, the evaluation of the accuracy of this approach with existing registration algorithms has been limited. For example, in SPM's unified segmentation method, cost‐function masking improves normalization accuracy when compared to not using it (Andersen, Rapcsak, & Beeson, 2010), although others have claimed that this approach does not outweigh the benefit of the initial tissue segmentation step (Crinion et al., 2007). In ANTs, constrained cost function masking (CCFM) limits the diffeomorphism to healthy tissue, leading the flow of the diffeomorphism in the lesion site to be interpolated. However, few studies have either examined the normalization performance of ANTs with CCFM (Kim, Avants, Patel, & Whyte, 2016) or compared it to SPM (Ripollés et al., 2012). It is worth noting that other methods for normalization of focal brain lesions include enantiomorphism (Nachev, Coulthard, Jäger, Kennard, & Husain, 2008), in which the lesioned area is replaced by the homologous, contralateral part of the brain. Because of the potential disadvantages for some types of lesions (e.g., for enantiomorphism, potential difficulties with bilateral lesions), we did not investigate these approaches.

Given the very limited number of studies validating recent normalization methods in patients with focal brain lesions (Ou, Akbari, Bilello, Da, & Davatzikos, 2014; Ripollés et al., 2012), we initially sought to apply the most widely used (SPM) and most accurate (ANTs) normalization methods for intact brains to lesioned brains. Here we found that ANTs‐based methods performed more accurately than SPM in terms of providing higher cross‐subject anatomical overlap, which is ultimately the goal of an accurate normalization. Yet, even in this case, normalization resulted in both suboptimal accuracy and accuracy biases between groups as a result of the warp from native to MNI space. To overcome these limitations, we next developed a staged approach to brain normalization that includes the iterative generation of an intermediate, cohort‐specific template. Based on the potentially large differences between any individual lesioned brain and the MNI template brain, the creation of an intermediate, cohort‐specific template allows for a series of graded, transitional optimization steps. The process of constructing an intermediate template has been introduced in past studies for increasing the fidelity of voxel‐based morphometry studies (Kim, Kim, & Jeong, 2017; Mak et al., 2011). This approach has primarily used the SPM‐based toolbox DARTEL (Ashburner, 2007) that creates a template by optimizing diffeomorphic‐based registration between images, though with fewer degrees of freedom and directionality constraints compared to the ANTs SyN method. Use of an intermediate template has also been introduced in the context of improving reproducibility in longitudinal studies of healthy individuals (Jacobacci et al., 2020; Tustison et al., 2017).

However, given that previous studies have shown the advantage of using ANTs over DARTEL (Klein et al., 2009), and the limited use of intermediate templates in lesion studies, we propose an ANTs‐based template solution for improved normalization. We demonstrate that including this intermediate, cohort‐specific template significantly improves normalization accuracy over current methods, reflected not only in the fidelity of the normalized anatomical images, but also in the localization of the blood‐oxygen‐level‐dependent (BOLD) signal in functional MRI data.

## 2 METHODS

To analyze and improve upon existing normalization methods in brains with focal lesions, we undertook a series of five experiments:

1. In Experiment 1, we created brains with virtual focal lesions to test SPM, ANTS, and the use of an intermediate, cohort‐specific template. Here lesion volumes from brain scans of individuals who suffered a stroke were inserted into brain images from healthy individuals to permit an unbiased estimation of registration error, as normalization accuracy can be compared across the virtual lesion images and the unaltered, healthy control brain images (Brett et al., 2001).
2. In Experiment 2, we validated the metrics and results derived from Experiment 1 in real brain data from stroke patients. Here we assessed normalization accuracy for a subset of the ATLAS dataset, an open source dataset of T1‐weighted structural brain MRI scans of stroke patients that includes corresponding manually traced lesion masks (Liew et al., 2018).
3. If an algorithm improves normalization accuracy within a set of brain scans from stroke patients, it should also reduce the difference in normalization accuracy between stroke and control brains. In Experiment 3 we utilized the metrics and results validated in Experiment 2 to compare normalizations in stroke patients and controls from a previous study (Gratton, Nomura, Pérez, & D'Esposito, 2012).
4. Greater normalization accuracy should improve the co‐registration of not only structural data, but also functional imaging data. In Experiment 4, we re‐analyzed data from stroke patients who underwent a functional MRI scan during the performance of a task in order to assess whether more accurate normalization improves localization of regions of interest derived from univariate statistics (Miller, Vytlacil, Fegen, Pradhan, & D'Esposito, 2011).
5. Given the increased use of functional connectivity analyses of brain imaging data, we sought to determine whether group functional connectivity results might differentially recover networks of brain regions following distinct normalization methods. For this exploratory experiment, we used resting‐state functional MRI (fMRI) data from a subset of stroke patients from Experiment 3 to probe the effect of normalization methods on localization of seed‐to‐voxel functional connectivity.

### 2.1 Experiment 1

#### 2.1.1 Dataset, imaging acquisition, and preprocessing

For the first experiment, we constructed virtual lesions using a combination of control and stroke data. Control data included a set of six healthy subjects from the LONI Probabilistic Brain Atlas (LPBA40) data at the Laboratory of NeuroImaging (LONI) at the University of Southern California/USC (Shattuck et al., 2008) (available online at https://resource.loni.usc.edu/resources/atlases-downloads/). Data acquisition was conducted after the approval of the local ethics committees and in accordance with the 1964 Declaration of Helsinki. Informed consent was obtained from all subjects. Acquisition parameters of the T1‐weighted images were as follows: 10–12.5 ms TR; 4.2–4.5 ms TE; 20° flip angle. We chose this subset based on the quality of the data and its manually labeled structures. Each T1‐weighted image consisted of 256 × 256 × 124 voxels with 0.86 × 0.86 × 1.5 mm^3^/voxel resolution. The T1‐weighted images were preprocessed according to existing LONI protocols to produce skull‐stripped brain volumes, then aligned to the MNI305 atlas (Evans et al., 1993) using rigid‐body transformation to correct for head tilt and reduce bias in the manual labeling process. In each of the six subjects, 56 structures were manually labeled in the MNI305A space of each subject's T1‐weighted image according to custom protocols using the BrainSuite software package (https://neuroimage.usc.edu/neuro/BrainSuite). Because of the MNI305 transformation, an additional registration step was needed to register the labels to their corresponding anatomical images in native/T1‐weighted space. Lesion masks were obtained from structural imaging data of six chronic stroke patients (Gratton et al., 2012). Informed consent was obtained from participants in accordance with procedures approved by the Committees for Protection of Human Subjects at the University of California, Berkeley and in accordance with the 1964 Declaration of Helsinki. We focused on this set of patients, as we wanted to include lesions of varying sizes and locations. Structural images were acquired on a 3‐Tesla Siemens MAGNETOM Trio MRI scanner using a 12‐channel head coil. An axial magnetization prepared rapid gradient echo 3‐D T1‐weighted sequence was used with the following specifications: 240 × 256 × 160 voxels,1 × 1 × 1 mm^3^/voxel resolution, repetition time TR = 2,300 ms, echo time TE = 2.98 ms, and flip angle = 9°. Lesions were drawn by co‐author HH on the native space of T1‐weighted images using the ITK‐SNAP software package (Yushkevich, Gao, & Gerig, 2016) and reviewed by neurologists AK and MD. Lesion size was calculated as the number of nonzero voxels and extracted using fslmaths (Jenkinson, Beckmann, Behrens, Woolrich, & Smith, 2012). The distribution of the lesion locations can be seen in Supporting Information, Figure S1.

To produce virtual lesions the six healthy LPBA40 data were combined with the six lesion masks from the stroke cohort to produce a total of 6 × 6 = 36 virtual lesion images. We used an affine registration to register the LPBA40 data to the lesion data. We then inserted lesions into the healthy images via the process described by Brett and colleagues (2001) to create abnormal, virtual lesioned images. To minimize potential intensity differences between the inserted lesion and the original healthy image, a scaling factor was applied to the abnormal image. To calculate the scale factor, we first computed the masked mean of the normal healthy image, divided by the masked mean of the abnormal image. For both the normal and abnormal images, the masked mean refers to the mean intensity of all voxels inside the brain but not in the lesion. The lesion region was then multiplied by this factor and inserted into the healthy image. Code is provided in the Supporting Information S1.

#### 2.1.2 Normalization

##### Methods

Three methods were used for normalization.

(1) SPM_us: SPM's unified segmentation (SPM_us: Ashburner & Friston, 2005): SPM's new unified segmentation method was chosen for three reasons: (a) Its prevalence in the stroke literature; (b) its open source code; and (c) its applicability in different types of lesions (e.g., bilateral lesions). SPM's unified segmentation normalizes images to MNI space by performing tissue matching based on tissue probability priors included with the SPM package (SPM12 Wellcome Department of Imaging Neuroscience, University College, London, UK, www.fil.ion.ucl.ac.uk/spm/, version 7771). This method is based on tissue matching and does not necessarily require spatial knowledge of the lesion. SPM12 was used in conjunction with MATLAB version 2019b (Mathworks, Natick, MA). Parameters were set to their defaults (number of Gaussians: two for gray matter, two for white matter, two for CSF, three for bone, four for other soft tissues, and two for air [background], bias regularization = 0.0001, warping regularization = 0, 0.001, 0.5, 0.05, 0.2). Code is provided in the Supporting Information S1.

(2) ANTs_mni: The diffeomorphic (SyN) method implemented by the Advanced Normalization Tools package‐ANTs (http://stnava.github.io/ANTs/, Avants et al., 2011) was chosen because it was previously identified as the most accurate method for normalizing healthy brains (Klein et al., 2009). ANTs implement a diffeomorphic‐based method, SyN, for registering images that allows nonlinear deformations between images to be parameterized with millions of degrees of freedom. This diffeomorphism‐based image registration framework utilizes geodesic/shortest‐path distances between points in space in order to maximize the desired similarity metric between images. Specifically, ANTs calculate an optimal diffeomorphism between images, as well as its inverse, thereby guaranteeing symmetry in the deformation (i.e., it is irrelevant which image is the source and target for registration). The gradient descent‐based algorithm works iteratively until convergence to find a diffeomorphism, split into two parts to maximize cross‐similarity between images.

SyN has been implemented in ANTs as a simple command (antsRegistrationSyN.sh). The antsRegistrationSyN.sh command applies, by default, rigid and affine transformations prior to the SyN transformation. The similarity metric used was cross‐correlation, the gradient‐descent step size was set to 0.25, and the number of iterations was set to 3. Otherwise, the parameters were set to the defaults. In the presence of lesions, preliminary work has shown that SyN works better when constrained cost function masking (CCFM) is used (Kim et al., 2016 and Figure S3). Like the original cost function masking (Brett et al., 2001), the CCFM approach calculates the mapping between images by considering all voxels except the ones in the lesion area. It then interpolates the missing values to complete the deformation. By definition, this approach requires a lesion mask that identifies the lesioned voxels (1 within the lesion, 0 everywhere else). In ANTs this step is incorporated via the –*x* option, followed by the inverse of the lesion mask—that is, 0 within the lesion, and 1 everywhere else. To transform the lesion masks to their inverse we used *fslmaths* with the –bininv option (Jenkinson et al., 2012). An example of code used for implementing SyN with CCFM is shown here (full code is provided in the Supporting Information S1):

fslmaths lesion.nii.gz –binv non_lesion

${ANTSPATH}antsRegistrationSyN.sh –d 3 –f t1mprage.nii.gz –m MNI_TEMPLATE.nii.gz –x non_lesion.nii.gz –o TRA

For the MNI‐152 image, we used the MNI152NLin2009cAsym template (Fonov et al., 2011). It is worth noting that instead of registering entire T1 images one can improve normalization accuracy by using skull stripped images (Klein et al., 2009). Due to potential variability in the accuracy of brain extraction when lesions are present, we chose to avoid this option in order to prevent discrepancies in normalization solely based on imperfect brain extraction. However, we noted no concerns visually when post hoc extractions using ANTs (command “antsBrainExtraction.sh”) were performed (data not shown). Thus, all results derived using the skull‐stripped versions of the images under consideration were virtually unchanged. Although outside the scope of the current work, we do provide code to successfully extract brain images from T1‐weighted data in the Supporting Information S1.

(3) ANTs_cohort: Generally, we use the term “ANTs_cohort” to describe the two‐step process of (a) constructing the cohort‐specific template, and (b) registering images to it using CCFM.

#### 2.1.3 Step 1

**FIGURE 1:**
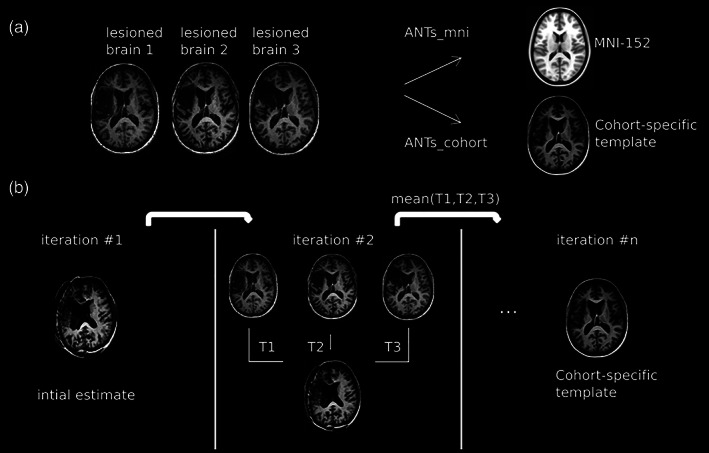
Overview of Step 1 in the ANTs_cohort pipeline in lesion patients. (a) For normalization we investigate two different methodologies, either (1) normalize the lesioned brains directly to the MNI‐152 template, or (2) construct a template representative of the subjects under investigation (cohort‐specific template/CST) and normalize the lesioned brains to the template (ANTs_cohort). (b) For the latter, the CST is constructed in iterative fashion using the T1‐weighted images from stroke patients. The mean image can be used as an initial estimate. At each iteration, the original images are warped to the CST using the SyN diffeomorphism transformations *T*_*i*_. The mean of these transformations is then applied to the template at the previous step to construct a new template. This process is repeated until convergence. In this way, the CST provides a representative morphology for the subjects under consideration—that is, it is “equi‐distant” from all the images. As a second step, the original images are normalized to the CST using constrained cost function masking (see main text)

A central theme in this article is the use of an intermediate, cohort‐specific template for improved registration. To do so, we begin by calculating the average of the structural images in the cohort to estimate an initial cohort‐specific template (CST), and then follow the template construction algorithm as implemented in ANTs (Avants et al., 2010). A schematic outline of template construction is as follows (Figure 1):

1. Register (transform) all images to the CST using SyN.
2. Average the newly transformed images to create a new CST.
3. Average all the transforms from Step 1 to create a single transform.
4. Apply the average transform from Step 3 to the CST to warp it toward the true mean shape.
5. Return to 1.

As mentioned previously, SyN treats both template and target symmetrically, thus guaranteeing that image features from the individual and the CST are used to drive the mapping throughout the optimization. In addition, the template method includes a template appearance optimization step (included in Step 2). This step is used to reduce both local tissue inhomogeneity and slowly varying distortions in tissue appearance. The CST in this step is updated using a gradient descent approach that maximizes the cross‐similarity between regional patterns within the CST. A subsequent update step (Step 4) incorporates the mean of the diffeomorphisms, thus modifying the CST to better match the included images (Avants et al., 2010). Here we used “buildtemplateparallel.sh” as implemented in ANTs (Avants et al., 2011). Minimum code for making a template given your anatomical images is the following:

${ANTSPATH}buildtemplateparallel.sh –d 3 –o output ${PATH_TO_T1s)/*.nii.gz

The gradient descent step size was set to 0.25, and the number of iterations of the previous process was set to 4. The process also includes an N4 bias field correction on the input images prior to template construction. This method of bias field correction iteratively remaps the intensities of the images by deconvolving them with a Gaussian histogram that represents the bias field corruption, followed by spatial smoothing using a *B*‐spline approximation algorithm (Tustison et al., 2010). We did not explore the use of brain‐extracted images to create a (brain extracted) template in order to be consistent with the ANTs_mni method, although we predict that this approach could provide more accurate normalization based on previous work applied to healthy data (Klein et al., 2010).

In this experiment, we created a template representative of virtual lesions and the control images from which they were derived. Per previous studies (Klein et al., 2010), demonstrating the utility of a template to generalize to new data requires the construction of a template that does not use the subjects' brains that are under consideration. Thus, we wanted to build a template that includes images of different brains but retains a *representative* morphology. To do so we constructed new virtual lesioned images by inserting the same lesions as used in the original dataset to three randomly chosen control subjects. These subjects were part of the LPBA40 dataset but different from the original set of healthy subjects used for evaluating normalization accuracy. A template was then constructed using these virtual lesion images and their corresponding normal images. We note that we chose not to use CCFM while constructing the template (i.e., when performing the registration of the images to the template at each iteration). While it is true that this approach might include some traces of lesion signal, this effect would be alleviated with the use of CCFM when we normalize the patients' data to the template in Step 2 of the ANTs_cohort process.

#### 2.1.4 Step 2

After the cohort‐specific template was created, this template space replaced the MNI‐152 space, and the previously described process for evaluating accuracy between pairs of virtual lesions was repeated. Importantly the CCFM approach is maintained even when projecting to the template space. Minimum code for the two steps combined is as follows (full code is provided in the Supporting Information S1):

# build template

${ANTSPATH}buildtemplateparallel.sh –d 3 –o output ${PATH_TO_T1s)/*.nii.gz

# perform registration

${ANTSPATH}antsRegistrationSyN.sh –d 3 –f t1mprage.nii.gz –m outputtemplate.nii.gz –x non_lesion.nii.gz –o TRA

# bring image to cohort‐specific template space

${ANTSPATH}antsApplyTransforms –d 3 –i t1mprage.nii.gz –r outputtemplate.nii.gz –t [TRA0GenericAffine.mat,1] –t TRA1InverseWarp.nii.gz –n Linear –float 1

##### Accuracy

To assess normalization accuracy for the different registration algorithms, we used each algorithm to nonlinearly register pairs of images—source (*l1*) and target (*I2*)—via a common space, as in Klein et al. (2009, 2010). We next applied the resulting nonlinear transformation *X*_I1⇝*space*_ (with nearest‐neighbor interpolation) to the corresponding linear source labels *L*_*I*1_ in order to produce warped source labels *L*_*I*1_*space*_. Then, using the (inverse) transformation of *X*_I2⇝*space*_, these labels were inversely warped to the target's *I*2 native space *L*_*I*1_*space*_*I*2_ and compared against the manual labels *L*_*I*2_ of image *I*2. This measure evaluates the performance of each algorithm by estimating the resulting cross‐participant overlap (which is the desired objective for normalization for fMRI purposes) instead of how well each image is aligned to the target space (Yassa & Stark, 2009). This process was conducted for all possible pairs of the 36 images. Using this process we compared the accuracy of different registration algorithms, SPM, ANTs_mni and ANTs_cohort. As described in the previous section, for each algorithm we used the appropriate transformation that resulted from the respective method (e.g., diffeomorphisms for ANTs) and the appropriate common space (template for ANTs_cohort and MNI‐152 for the rest).

To evaluate normalization accuracy, the Jaccard coefficient between the warped labels L_I1_space_I2_ and the manual labels L_I2_ (Jaccard, 1912) is defined as their volume of intersection divided by the total volume:

Jaccard coefficient = |*L*_*I*1_*space*_*I*2_ ∩ *L*_*I*2_|/|*L*_*I*1_*space*_*I*2_ ∪ *L*_*I*2_|

Because it can directly assess the effect of the registration method on each of the 56 manually defined anatomical regions, this metric captures normalization accuracy more sensitively than previously reported low‐level metrics (such as the root mean square distance; Rohlfing, 2012). The final registration accuracy number reported is the mean of the Jaccard coefficient over all 56 manually defined regions in the registration space.

For this experiment, we compared normalization accuracy between SPM, ANTS_mni and ANTs_cohort using the virtual lesion data. Accuracy for each lesioned brain was compared with accuracy for the unlesioned brain from which it was derived, treating the latter as ground truth. Thus, we evaluated the absolute *difference* between the Jaccard overlap in the virtual lesion images and the Jaccard overlap in their corresponding unlesioned images. Specifically:

1. For each registration method, we ran the Jaccard overlap for the virtual lesion brains.
2. For the same registration method, we repeated Step 1 for the healthy brains from which the virtual lesion brains were derived.
3. We calculated the absolute difference in accuracy between the two.

The absolute difference provides an unbiased estimate of normalization accuracy relative to a “ground truth,” regardless of the particular algorithms each registration method employs. Higher values demonstrate that a method performs more poorly for lesions compared to the unlesioned data. Code for the Jaccard index is provided in the Supporting Information S1.

### 2.2 Experiment 2

#### 2.2.1 Dataset, imaging acquisition, and preprocessing

For the second experiment, we used stroke data drawn from the ATLAS database and assessed normalization using the ANTs_mni and ANTs_cohort methods outlined in Experiment 1. For our analyses, we used part of the ATLAS dataset, an open source dataset of T1‐weighted structural brain MRI scans of stroke patients with manually traced lesion masks available both in native and MNI‐152 space (https://www.icpsr.umich.edu/web/ICPSR/studies/36684/summary, Liew et al., 2018). For purposes of evaluating normalization accuracy we used the T1‐weighted data and their lesion masks in native space. Data acquisition was approved by local ethics committees and conducted in accordance with the 1964 Declaration of Helsinki. Informed consent was obtained from all subjects. All 229 scans of the ATLAS dataset were completed on multiple 3‐Tesla MRI scanners at 1 mm isotropic resolution. Acquisition details are in Liew et al., 2018. Manual segmentations on left and right hippocampus were conducted in these subjects by trained experts; thus we only used this subset of 30 stroke patients. All manual segmentations are available for download (https://github.com/npnl/Hippocampal_Segmentation) (Zavaliangos‐Petropulu et al., 2020). Because the available segmentations were provided in MNI‐152 space (compared to the T1‐weighted and lesion data that were provided in both native and MNI‐152 space), we used SPM12 (version 7771) to inverse normalize them to native space (code is provided in the Supporting Information S1). This choice does not impact the results, as in this section we are only comparing the ANTs methods. There was no further preprocessing other than that included in the construction of the template.

#### 2.2.2 Normalization

##### Methods

We used the ANTs_mni and ANTs_cohort methods for evaluating normalization. To ensure that our results for the latter method were not biased, we used a template based on stroke patients who were not part of the ATLAS data under investigation, as described in Experiment 3.

##### Accuracy

Similar to Experiment 1, we considered all possible pairs of the 30 patients using the Jaccard overlap as a measure of accuracy. The final Jaccard score reported for each method was the average score from the left and right hippocampal segmentations. One subject (and all its associated pairs) had to be removed from the final results due to failed registration.

### 2.3 Experiment 3

#### 2.3.1 Dataset, imaging acquisition, and preprocessing

In this experiment, we sought to determine whether the cohort‐specific template method reduced differences in normalization accuracy between stroke patients and healthy controls. Eleven patients with focal lesions due to stroke (age range 51–84 years, time post stroke range 7 months to 12 years), as well as 6 healthy controls (age range 22–36), were included. A description of the data can be found in (Gratton et al., 2012). Informed consent was obtained from participants in accordance with procedures approved by the Committees for Protection of Human Subjects at the University of California, Berkeley. Acquisition details for all the patients were identical to the patient cohort used for creating virtual lesions in Experiment 1. Acquisition details for the healthy controls were identical to those of the stroke patients. We chose 11 patients due to their variety of lesion locations and sizes. Of the 11 patients, 6 patients had left middle cerebral artery (MCA) strokes and 5 patients had posterior cerebral artery (PCA) strokes. An additional set of 6 right MCA patients (age range 36–63 years, time post stroke range 3 months to 9 years) was used in order to validate the accuracy differences between patients who were part of the template and patients who were not part of the template. Lesions were drawn by co‐author HH on the native space of T1‐weighted images using the ITK‐SNAP software package (Yushkevich, Gao & Gerig, 2016) and reviewed by neurologists AK and MD. Lesion size was calculated as the number of nonzero voxels and extracted using fslmaths (Jenkinson et al., 2012). The distribution of the lesion locations can be seen in the Supporting Information Figure S1.

Besides the preprocessing involved in the template we also obtained the skull‐stripped images from the patient and control data (using “antsBrainExtraction.sh” with default settings). This was done because we calculated mutual information (a measure of normalization accuracy‐see below) only within the brain voxels in order to disregard potential differences introduced by voxels of no interest (e.g., neck, skull, and so on). Thus we ultimately compared normalized mutual information between brain voxels of patient and control data and the skull stripped versions of the MNI‐152 template (for ANTs_mni) and the cohort‐specific template (for ANTs_cohort). The MNI‐152 brain image is publicly available, whereas we performed “antsBrainExtraction.sh” to skull strip the cohort‐specific template. For the patient data gray matter, white matter and cerebrospinal fluid (CSF) segmentations were also obtained via the “antsCorticalThickness.sh” pipeline and used in Experiment 5 to denoise functional data.

#### 2.3.2 Normalization

##### Methods

We used ANTs_mni and ANTs_cohort as our registration methods. For the latter, a template representative of both the stroke patients and controls was constructed using the iterative process described in Experiment 1. As a result, this template captures the “average” morphology of all the included images in order to reduce normalization accuracy differences between groups.

##### Accuracy

Because of the lack of manual segmentations for this cohort, we used normalized mutual information (NMI) to define accuracy. For a source image warped to a common space, this measure captures the similarity of the warped image to the common space (either MNI or cohort‐specific template, depending on the case). This metric is not sensitive to landmark displacement differences, thus rendering its use inappropriate for comparing between methods. However because (a) both ANTs_mni and ANTs_cohort as part of the ANTs suite have been shown here and in other studies (Klein et al., 2009, 2010) to produce anatomically meaningful image registrations and (b) because the only parameter changing between the two methods is the common target space, we used normalized mutual information for comparisons between the two methods. For an image *I*_*1*_ its entropy is defined as $H\left(I_{1}\right)=-\sum_{a\in I_{1}}P\left(a\right)\log\left(P\left(a\right)\right)$. The normalized mutual information between two images *I*_1_, *I*_2_ is then defined as *NMI*(*I*_1_, *I*_2_) = (*H*(*I*_1_) + *H*(*I*_2_))/*H*(*I*_1_, *I*_2_) where *H*(*I*_1_, *I*_2_) is the joint entropy (Nam et al., 2008). If one replaces *I*_1_ with the warped images to a common space and *I*_2_ with the image of the space per se (either MNI or template), one obtains a measure of similarity between the target image and the warped image. Intuitively, the higher the normalized mutual information, the greater the similarity between images *I*_1_, *I*_2_. For the ANTs_mni and ANTs_cohort methods, we assessed normalized mutual information differences in MNI space and the cohort‐specific template space, respectively, between patients and healthy controls only within the brain voxels—that is, *I*_*1*_ was the skull‐stripped image for each patient and healthy control, and *I*_2_ was either the skull‐stripped MNI‐152 template (for ANTs_mni) or the skull‐stripped cohort‐specific template (for ANTs_cohort). For validation, we compared the normalized mutual information between stroke patients who were not part of the template and the stroke patients and healthy controls who comprised the template. Code is provided in the Supporting Information S1.

### 2.4 Experiment 4

#### 2.4.1 Dataset, imaging acquisition, and preprocessing

To determine whether a cohort‐specific template improves the identification of univariate brain activations in prespecified regions of interest, we evaluated five individuals (ages 44–72 years) with lateral prefrontal cortex lesions due to stroke who participated in a face‐scene functional localizer experiment. Additional details can be found in Miller et al., 2011. Informed consent was obtained from participants in accordance with procedures approved by the Committees for Protection of Human Subjects at the University of California, Berkeley and in accordance with the 1964 Declaration of Helsinki. Images from one individual were discarded due to an issue with corrupted DICOM headers. T1‐weighted anatomical scans were acquired from a Varian INOVA 4‐Tesla scanner using a gradient‐echo multislice sequence (TR = 200 ms, TE = 5 ms, 256 × 256 × 128 voxels with 0.875 × 0.875 × 1 mm^3^/voxel). Lesions were manually segmented on the native T1 space by co‐author HH using ITK‐SNAP (Yushkevich et al., 2016) and inspected by neurologists AK and MD. The distribution of the lesion locations can be seen in the Supporting Information, Figure S1. Besides the preprocessing involved in the template, for better registration of functional to anatomical data, we also obtained the skull‐stripped images from the T1‐weighted data using the ANTs command “antsBrainExtraction.sh” with default settings.

Functional images were acquired from the same scanner during a localizer task in which patients viewed 16‐s blocks of faces or places, interleaved with blocks of fixation (Miller et al., 2011). Functional images were collected using a gradient‐echo‐planar sequence (336 time points repetition time TR = 2,000 ms, echo time TE = 28 ms, matrix size = 64 × 64, field of view FOV = 22.4 cm). Each functional volume consisted of 18 3.5 × 3.5 × 5‐mm‐thick axial slices with a 0.5‐mm gap between each slice. For preprocessing, functional volumes were slice time corrected, motion corrected using SPM12 v7771 and registered to anatomical, brain‐extracted images using the ANTs “antsRegistrationSyNQuick.sh” command with the “‐a” (affine transformation) option. We then used AFNI (Cox, 1996) to identify brain responses to each stimulus presentation. Each block of the task was modeled as a 16‐s epoch and convolved with a canonical hemodynamic response function. Block‐related effects were estimated for each subject using a fixed‐effects general linear model. This was implemented in AFNI's function *3dDeconvolve*. Linear contrasts were computed to isolate voxels significantly activated in face versus baseline. See Miller et al., 2011 for more details.

#### 2.4.2 Normalization

##### Methods

Group level statistics were conducted in the cohort‐specific template space as well as the MNI‐152 space. To do so the functional images were either mapped to MNI‐152 space using the transformations from the brain extracted images to MNI‐152 space (ANTs_mni method) or to the previously defined cohort‐specific template space using the transformation from the brain extracted images to the cohort‐specific template space (ANTs_cohort method). For the latter, a template consisting of the T1‐weighted images was constructed using the same process as described in Experiments 1–3. A statistical contrast “face greater than baseline” isolated significantly activated voxels that responded to faces. We thresholded the resulting t‐maps at *p* < .05 cluster size‐corrected (uncorrected threshold of *p* < .01 followed by cluster size correction of 30) in order to extract meaningful activations.

##### Accuracy

We then examined the overlap between these maps and regions of interest in the extrastriate cortex, namely the left and right fusiform area (FFA). The boundaries of these regions of interest were determined by an SPM‐based second level analysis in healthy participants, as described in Miller et al., 2011. Briefly four healthy controls underwent the same experimental condition (blocks of faces or scenes interleaved with fixation blocks). The contrast of face versus baseline was used to define the FFA clusters. Each block of the task was convolved with a canonical hemodynamic response function and a subject‐specific fixed‐effects model was used to identify face‐related effects. Based on the resulting group map at *p* < .05 cluster size‐corrected (uncorrected threshold of *p* < .01 followed by cluster size correction of 30), the 49 most activated voxels in the left and right FFA were isolated and further used as a region of interest (ROI) for our analysis. Accuracy was obtained as the overlap between the thresholded statistical maps and these regions of interest. Mathematically, this quantity was defined as the number of shared voxels between the functional map and the anatomical regions of interest, divided by the number of voxels in the region of interest. Overlap was quantified using a leave‐one‐out method in which, for each iteration, one subject was left out of the analysis and the remaining subjects were used to calculate the group level map. In the case of the ANTs_cohort method, because these regions of interest were defined in MNI‐152 space, we used a mapping from cohort‐specific template to MNI to bring the anatomical regions to cohort‐specific template space. This mapping was obtained by applying the SyN diffeomorphism registration used to register the cohort‐specific template to the MNI‐152 template. For clarity, visualization of the statistical maps and their overlap was restricted to an anatomically defined region of interest of the fusiform cortex derived from the Brainnetome atlas (Fan et al., 2016).

### 2.5 Experiment 5

#### 2.5.1 Dataset, imaging acquisition, and preprocessing

Lastly, we turned our focus to the effect normalization can have on functional connectivity MRI data. For this experiment, we used a subset of the left middle cerebral stroke patients from Experiment 3. In addition to the structural data described in Experiment 3, functional images were collected on a whole‐body 3‐T Siemens MAGNETOM Trio MRI scanner using a 12‐channel head coil. Ten minutes of EPI data were obtained for these stroke patients (300 time points, TR = 2,000 ms, TE = 30 ms, 28 3.3 mm thick axial slices, matrix size 128 × 128).

Regarding preprocessing, resting state functional images were slice‐time corrected and coregistered to the structural images (using “antsRegistrationSyNQuick.sh”). They were then normalized to a common space using the transformation obtained from ANTs_mni (in case of MNI) or ANTs_cohort (in the case of the cohort‐specific template) as described in Experiment 3. We used a Gaussian smoothing kernel of 8 mm full‐width at half‐maximum (FWHM). To obtain functional connectivity data, we used the Conn toolbox (Whitfield‐Gabrieli & Nieto‐Castanon, 2012). For denoising the time course data, five principal components were extracted from the white matter and CSF. These components along with the six motion parameters were used in a regression to denoise the time courses (Behzadi, Restom, Liau, & Liu, 2007). The time courses were then high pass filtered within the range [0.009 inf], despiked, and detrended.

#### 2.5.2 Normalization

##### Methods

As previously, the denoised functional images were then mapped to MNI‐152 space using the transformations from the ANTs_mni method or to the previously defined cohort‐specific template space using the transformation provided by the ANTs_cohort method. For the latter, a template consisting of the T1‐weighted image of the left middle cerebral artery stroke patients was constructed using the same process as described in Experiments 1–3. Results were unchanged when using the same template as in Experiment 3—that is, one not generated solely from the subset of patients examined here.

##### Accuracy

We quantified significant functional connectivity from the right FFA to the rest of the brain (seed‐to‐voxel connectivity) using the two normalization methods. We chose this seed because (Andersen et al., 2010) it lies in the contralesional hemisphere, thus minimizing the presence of noisy correlations resulting from the presence of the lesions in the left hemisphere, and (Ashburner & Friston, 1999) this area should have relatively intact connectivity to the left hemisphere, as the patients under investigation had MCA lesions that spared the temporal and occipital lobes. Statistical maps of seed‐to‐voxel connectivity were thresholded at *p* < .01 followed by cluster size correction *p* < .05. Because we anticipated that successful normalization should recover bilateral resting state networks that include the FFA, we compared the number of contralateral (in this case, left‐sided) voxels recovered by the two methods using a leave‐one‐out method (i.e., by removing one subject at the time and calculating the size of the cluster using the remaining subjects). Larger numbers of (clustered) contralateral voxels imply better recovery of resting state networks due to better co‐localization of connectivity between different patients.

### 2.6 Statistics

In Experiment 1, for comparison of normalization accuracy between methods we used a nonparametric Kruskal–Wallis ANOVA due to the non‐normal distribution of the data, with Bonferroni correction for the number of post hoc tests. Post hoc Wilcoxon rank sum tests with *p* < .05 (corrected) were deemed statistically significant. A nonparametric paired Wilcoxon test was used to compare normalization accuracy between the two methods in Experiment 2. For comparisons of normalization accuracy between different groups, we used a one‐way (unbalanced) analysis of variance (ANOVA). We chose not to report post hoc tests due to the limited sample. Correlations between normalization accuracy and lesion size represent Pearson's *r* values (although due to the limited sample size they remain exploratory). Comparisons between correlations were performed using Steiger's statistic. Second‐level FFA activation and connectivity statistical considerations are described in their respective sections.

## 3 RESULTS

### 3.1 Comparison of different normalization methods in virtual lesion brains

Here, we explored the impact of two widely used normalization methods—ANTs normalization to MNI‐152/ANTs_mni, and SPM's unified segmentation/SPM_us—alongside a newly introduced approach, ANTs_cohort, that involves normalization to a cohort‐specific template (Methods). We first tested the accuracy of these algorithms on a set of virtual brains in which lesions from patients were manually inserted into healthy brains (Methods and Figure S2). Given the enormous resources required to obtain brain scans from a large number of stroke patients, the creation of such virtual lesions allows for large sample sizes while also permitting us to calculate the performance of each method on lesion data in the absence of unlesioned data before the lesion. Specifically, performance of each method was assessed using the label overlap of manually segmented regions between pairs of images in a common space. For each method, the accuracy on the virtual lesion data was subtracted (absolute difference) from the accuracy on the ground truth data, resulting in an accuracy measure that takes into consideration the ground truth performance of the method on healthy brains (Methods).

**FIGURE 2:**
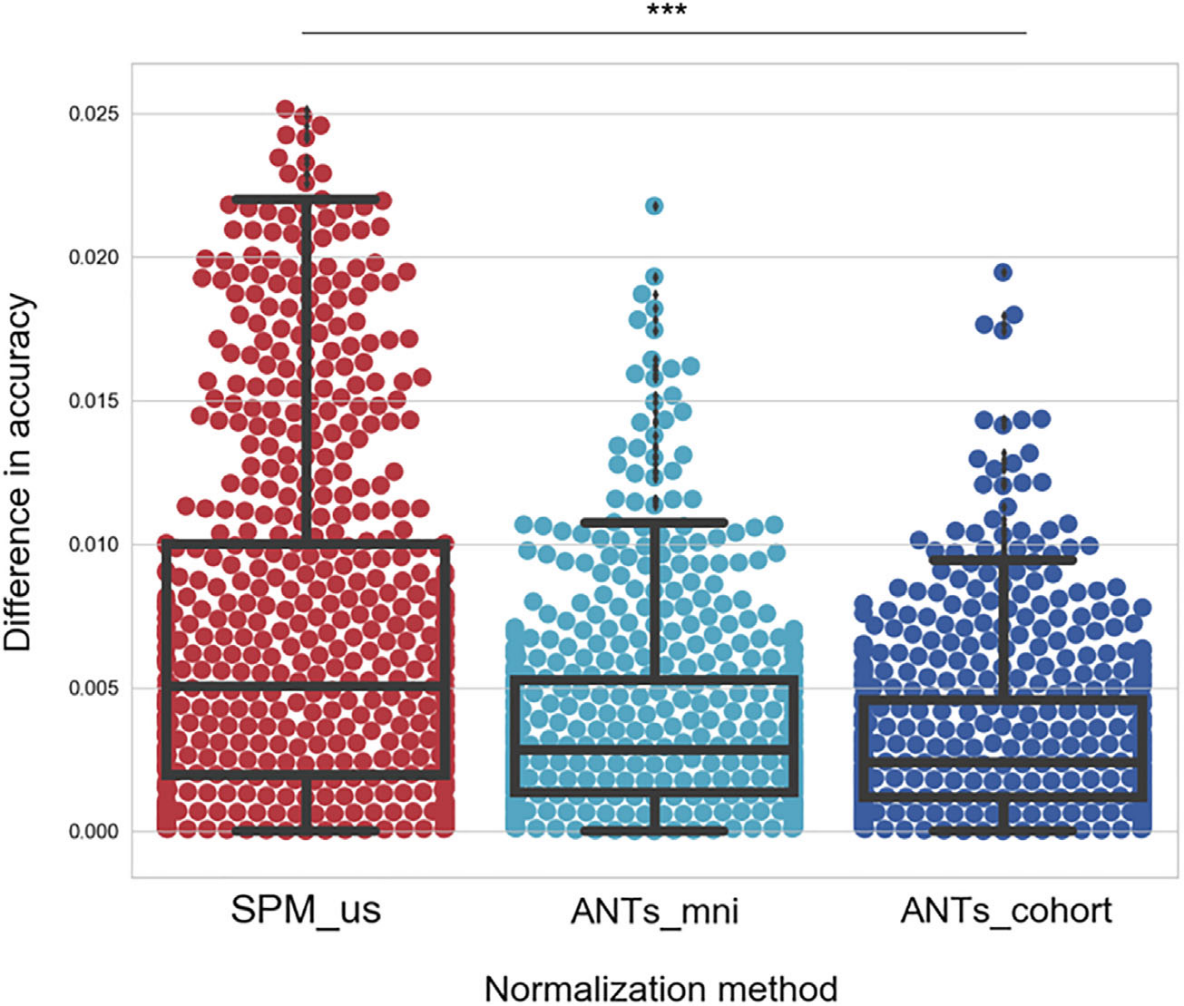
Experiment #1: Normalization accuracy in virtual lesion brains. By combining brain data from healthy individuals with lesion segmentations from stroke patients, virtual lesion images were created to evaluate normalization accuracy. For each registration algorithm, its accuracy in terms of Jaccard coefficient was assessed for pairs of images that were both registered to a common space (MNI‐152 or a cohort‐specific template). The *y*‐axis presents the absolute difference between normalization accuracy of virtual lesion images and their corresponding unlesioned images. Whiskers represent the 1.5 inter‐quartile range (IQR). Horizontal lines inside the boxplots represent median values. ***p < .001 Kruskal–Wallis ANOVA

We first noted that a large number of virtual brain normalizations using SPM failed (approximately 38%) due to the presence of two large MCA lesions used for producing virtual data. This finding forced us to restrict the number of pairs that ended up in the final comparison. In contrast, both ANTs methods provided results for all the pairs. For the remaining pairs we found that the normalization accuracy was different for different methods (Kruskal‐Wallis ANOVA chi‐squared[2,1980] = 117.5, *p* < .001, Figure 2), Specifically the normalization accuracy measured by the difference in Jaccard coefficient between virtual lesions and their corresponding control brains was smaller when using ANTs_mni compared to SPM (ANTs_mni vs. SPM_us difference in means = 0.0031, Wilcoxon test *p* < .001, Bonferroni corrected for three comparisons). These results show that the normalization error is greater when using SPM's unified segmentation method compared to the ANTs_mni method—that is, the SPM normalization is more strongly disrupted by the presence of lesions.

Although the ANTs‐based method is less disrupted by the presence of lesions, it is constrained by the fact that the MNI‐152 space is derived from healthy individuals. There are inherent difficulties in normalizing lesioned brains to MNI space, since this process involves transforming abnormal brain images to match a healthy brain template. An alternative approach to potentially improve the normalization accuracy is to utilize a cohort‐specific template rather than the MNI‐152 template, an approach that has been previously proposed for voxel based morphometry studies (Kim et al., 2017), longitudinal studies (Tustison et al., 2017), and improving registration for diffusion imaging data (Jacobacci et al., 2020). A cohort‐specific template incorporates the morphology of all patients' brain images in the particular study, allowing a more powerful and precise normalization of individual brains to a template space (Methods). To that end, when we normalized the virtual lesion data to a cohort‐specific template (ANTs_cohort), we found higher normalization accuracy compared to ANTs_mni that was of borderline significance (ANTs_cohort vs. ANTs_mni difference in means = 0.0005, Wilcoxon test *p* = .0561 Bonferroni corrected, Figure 2). The difference between the two methods was likely smaller compared to the difference between ANTs_mni and SPM_us because of the intrinsic difficulty in creating a template that equally represented both the virtual lesion brain and the healthy brain from which it originated (as this is how the difference score is calculated). Despite this issue, a template with a representative morphology of the stroke data under investigation resulted in improved normalization accuracy.

An additional method used for improving normalization via an intermediate template is DARTEL (although with different parameterization compared to ANTs). Thus, we also compared the aforementioned methods to DARTEL. We found that SPM_us, ANTs_mni and ANTs_template performed better compared to DARTEL in our virtual lesion data. Thus, we decided not to use this method further (Figure S4). In the next section, we turn to real stroke data to verify that the differences are not an artifact of the virtual lesion data.

### 3.2 Comparison of different normalization methods in real stroke data

**FIGURE 3:**
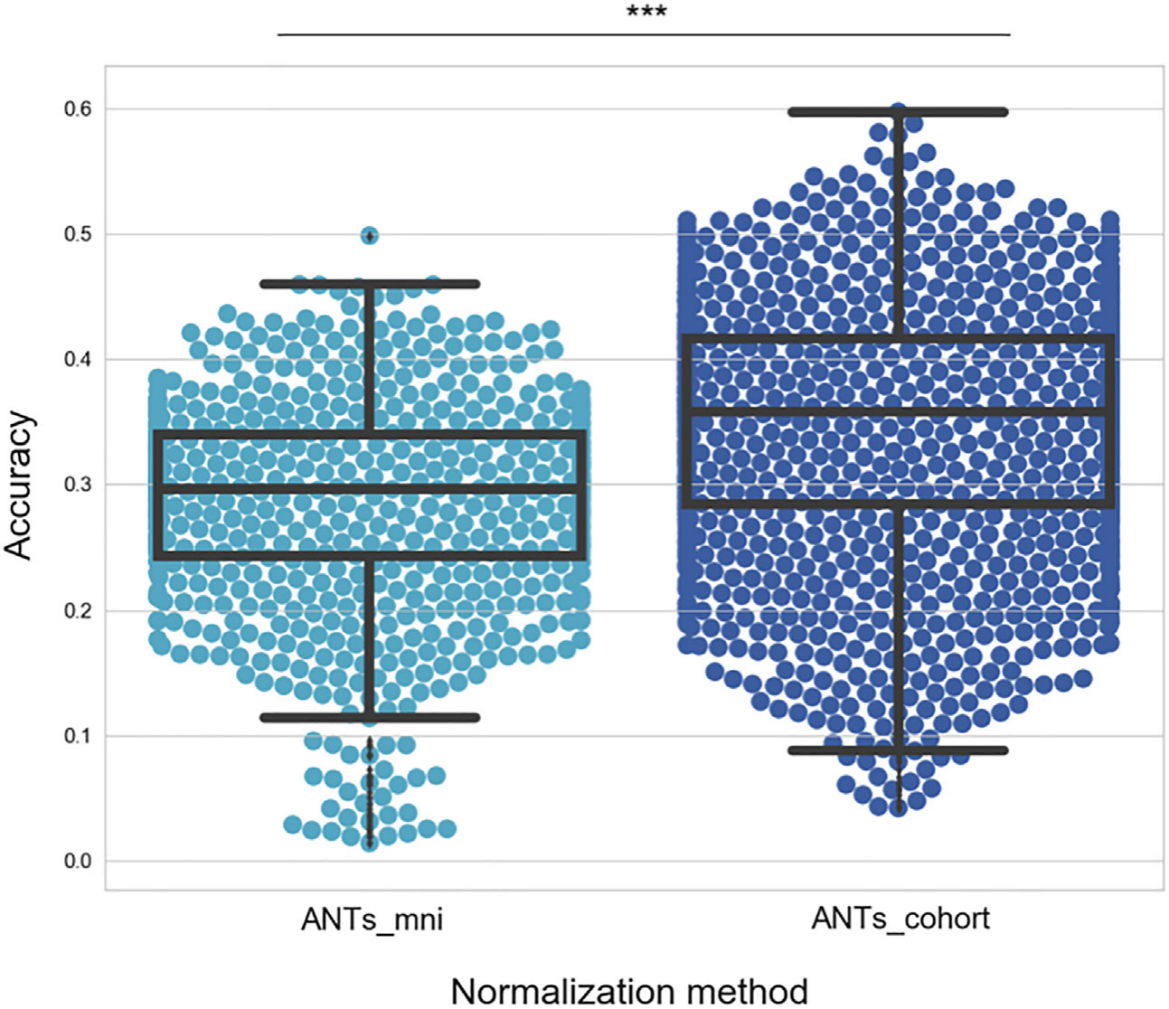
Experiment #2: Evaluating normalization accuracy in stroke patients with manual hippocampus segmentations. Normalization accuracy was defined between pairs of images using the overlap between the warped manual labels of the source image and the original labels of the target. The ANTs_cohort method is more accurate than the ANTs_mni method. ***p < .001 significance of a paired Wilcoxon test. Whiskers represent the 1.5 IQR. Horizontal lines inside the boxplots represent median values

Next, we assessed these normalization methods with real stroke data. To be consistent, we used the same accuracy measure as for the virtual lesion brains presented in Section 3.1, but without the ground truth accuracy (as there is no prelesion data). For this experiment, we evaluated a stroke patient dataset that included manual segmentations of left and right hippocampus (Methods and Figure S2). We found that the normalization accuracy improved when using a stroke cohort‐specific template compared to ANTs_mni (ANTs_cohort vs. ANTs_mni difference in means = 0.0526 paired Wilcoxon test *p* < .001 Figure 3). SPM_us was not tested in this case as it was used to bring the hippocampal segmentations to native space.

### 3.3 Comparison of normalization across different groups

The goal of normalization is to allow group‐level analyses to be performed, usually as comparisons between patient groups, or between patient groups and healthy controls. Any differences in normalization accuracy between groups under comparison could bias results regarding group differences found in consequent imaging data analyses. Because the MNI‐152 template consists of healthy brains, standard normalization to MNI‐152 space might be particularly prone to this bias. Specifically, normalization to MNI‐152 space might be more accurate for healthy controls, or certain patient groups with less abnormal pathology, compared to others with highly abnormal characteristics.

For this experiment, we examined whether our template method can minimize these potential between‐group accuracy biases. We used a dataset consisting of individuals with left middle cerebral artery (MCA) and posterior cerebral artery (PCA) strokes, and healthy controls (Methods). We chose this dataset because of the variety in lesion morphology and location, as well as the inclusion of healthy controls. Because no manually labeled regions were available for this cohort, we assessed normalized mutual information as a measure of normalization accuracy. The use of this metric is appropriate provided that only within‐methods comparisons are evaluated (Methods and Figure S2).

**FIGURE 4:**
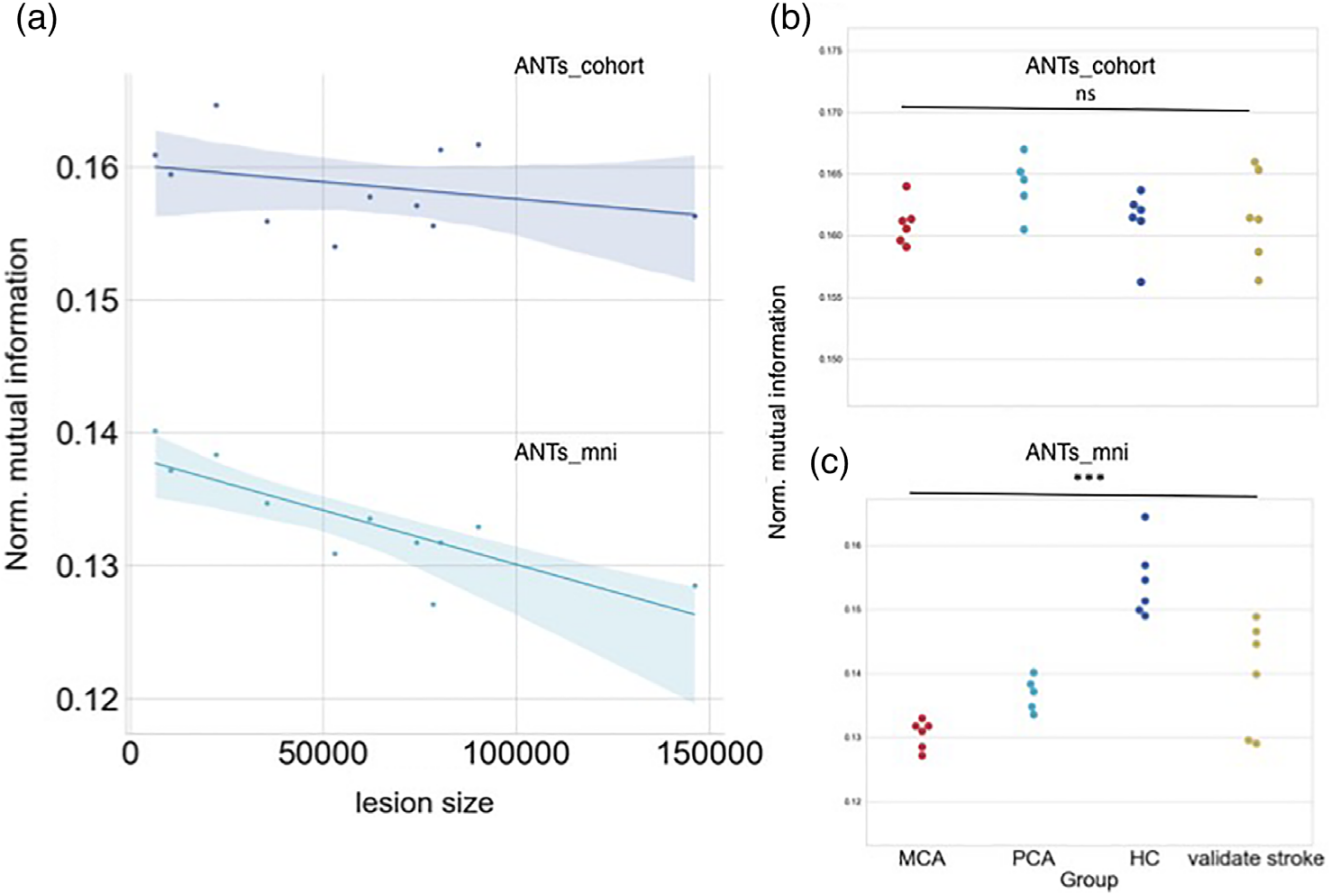
Experiment #3: Group differences in normalization accuracy. Normalization accuracy was assessed in healthy controls, patients with middle cerebral artery (MCA) or posterior cerebral artery (PCA) strokes, and a third set of patients with strokes in various locations for validation. (a) For the standard ANTs_mni method, normalization accuracy worsens when lesion size (in number of voxels) increases (lower line). For a cohort‐specific template, normalization accuracy shows less dependence on lesion size, and normalized mutual information (NMI) is greater overall (upper line). (b) No differences in normalization accuracy were found when a cohort specific template was used, even for stroke subjects who were not included in the template. (c) When ANTs_mni was used, normalization was most accurate for healthy controls compared to stroke patients. ***p < .001; n.s. = nonsignificant (one‐way ANOVA). Shaded regions around the correlations represent 95% confidence intervals

Using the ANTs_mni method, there was a dependence of normalization accuracy on lesion size, where normalization was less accurate in patients with larger lesions (Pearson's *r* = −0.828, *p* = .0016) (Figure 4(a)). Second, normalization accuracy was significantly worse in patients compared to controls (one‐way ANOVA *F* (2,19) = 19.553, *p* < .001, difference in means (patients all vs. healthy controls = 0.0187) (Figure 4(c)).

Given these results, we hypothesized that the cohort‐specific space would result in similar normalization accuracies between patient groups, and between patients and healthy controls. With this cohort specific template, the dependence of normalization accuracy on lesion size (*r* = −0.3242, *p* = .3307) was smaller for the cohort method compared to the dependence when normalizing directly to MNI space (*r* = −0.8285, *p* = .0016) (Steiger's *z*‐statistic for comparing correlations = 2.421, *p* = .008) (Figure 4(a)). In addition, there were no significant differences in normalization accuracy between stroke patients and healthy controls (*F*(3,19) = 1.48 *p* = .2515 (n.s.), Figure 4(b)).

### 3.4 Effect of normalization method on functional imaging

In this next set of analyses, we show the effect of normalization using a cohort‐specific template on univariate and functional connectivity analyses of fMRI data.

#### 3.4.1 Univariate analysis

**FIGURE 5:**
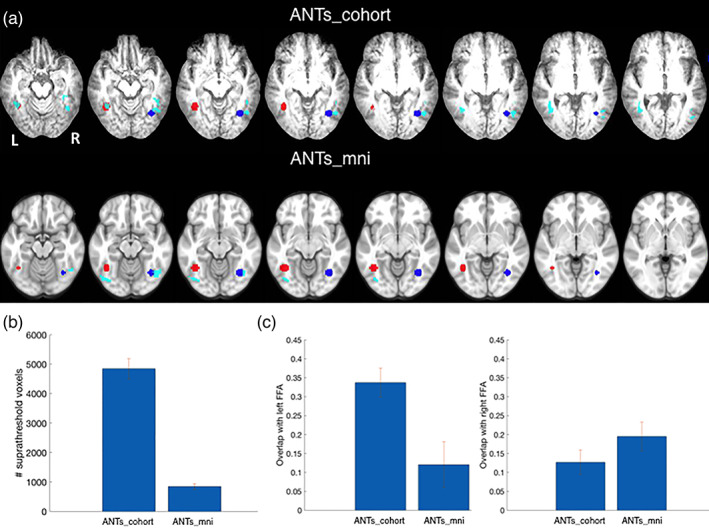
Experiment #4: Effect of normalization on univariate statistics of fMRI data. Data is presented from four patients with frontal lesions due to stroke during performance of a face‐processing task. (a) Thresholded statistical maps within fusiform gyrus (light blue) showing task activation for ANTs_cohort method (top row) and for the ANTs_mni method (bottom row). The right and left fusiform face regions of interest (see method section) are shown in blue and red on the cohort‐specific and MNI‐152 templates. (b) Total number of suprathreshold voxels within fusiform gyrus for the ANTs_cohort and ANTs_mni methods. Note the greater number of voxels when the ANTs_cohort method was used. (c) This panel shows the overlap with the left and right FFA ROIs for the ANTs_cohort and ANTs_mni methods. For panels (b) and (c) mean and *SE* are shown over different leave‐one‐out iterations. Note the greater overlap of activation with the left FFA ROI in the ANTs_cohort template as compared to the ANTs_mni template

In this task, four patients with frontal lesions viewed face and scene images while BOLD images were obtained (Methods). We only focused on the face condition; this was a primary analysis of interest in the original article, given the goal of evaluating visual cortex activations in the presence of lesions (Miller et al., 2011). Contrasts of the beta values for the face condition versus baseline were obtained and we measured the overlap of the resulting statistical maps with the left and right fusiform face area (FFA) regions using a leave one out methodology. Significant voxels were identified using both methods at *p* < .01 uncorrected followed by cluster size correction at *p* < .05. We observed a higher overlap in the left FFA, and more active voxels in fusiform gyrus, when using the ANTs_cohort method compared to the ANTs_mni method, while ANTs_cohort and ANTs_mni were more similar in the right FFA (Figure 5 and Table 1). This result indicates that different normalization methods can potentially shift the loci of significant functional activations, and suggests that higher normalization accuracy may lead to better localization of the significant effects within regions of interest.

**TABLE 1:** Total number of suprathreshold fusiform gyrus voxels as well as overlap of statistical maps obtained from Experiment #4 representing FFA responses with FFA regions of interest (left and right FFA)

Normalization method	Suprathreshold fusiform gyrus voxels	Overlap left FFA	Overlap right FFA
ANTs_cohort	4,845.8 (346.7)	0.3373 (0.0383)	0.1267 (0.0328)
ANTs_mni	846.5 (99.4)	0.1207 (0.0603)	0.1949 (0.0382)

*Notes*: In the first row, the functional data were normalized to the MNI‐152 space. In the second row, the functional data were normalized to a cohort‐specific template space. Mean and *SE* are shown over different leave‐one‐out iterations.

#### 3.4.2 Functional connectivity analysis

**FIGURE 6:**
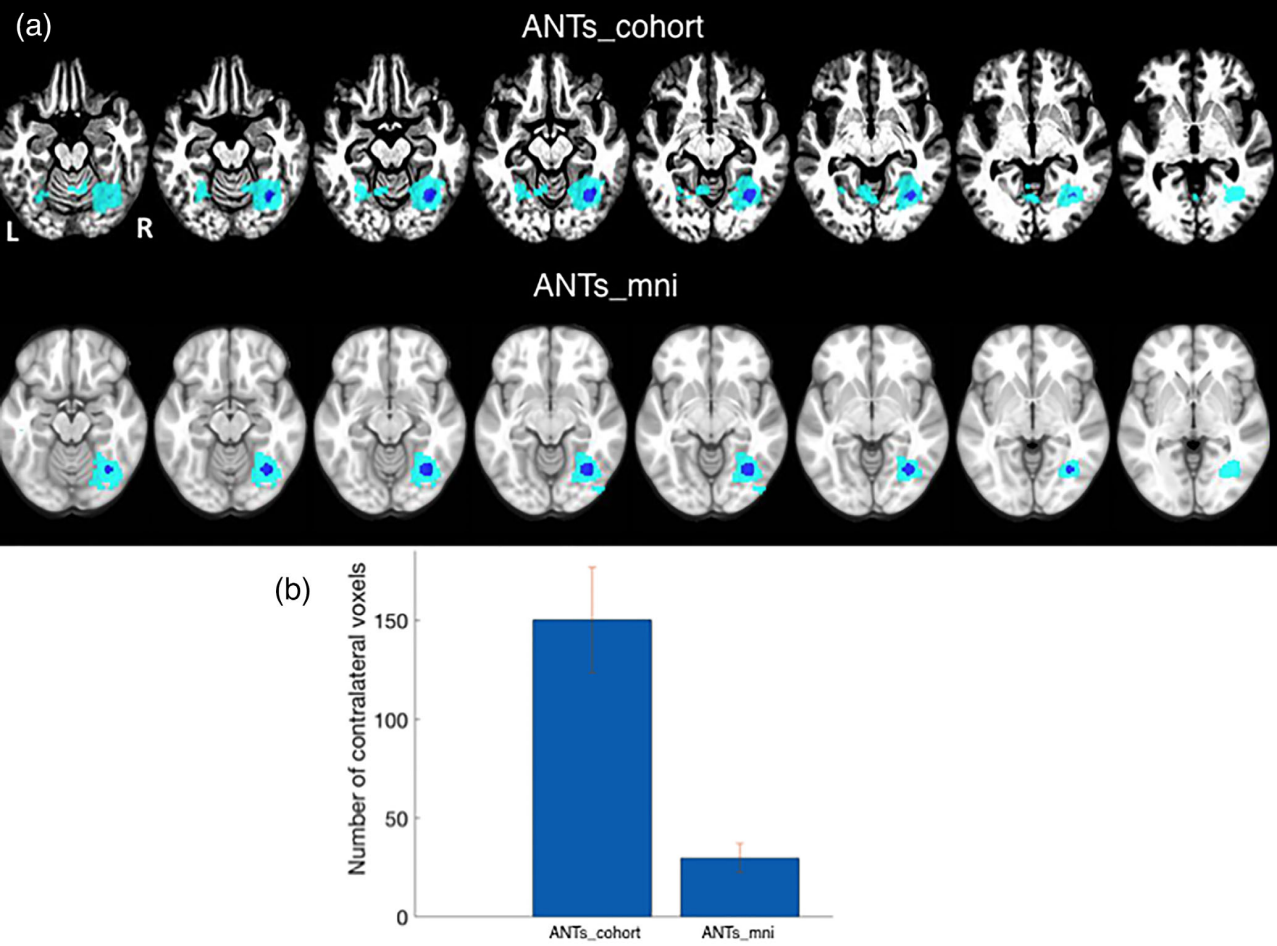
Experiment #5. Effect of normalization on seed‐to‐voxel connectivity analysis of fMRI data. (a) The right fusiform face area was used as a seed (shown in blue). Brain regions functionally connected to this seed are shown for the ANTs_cohort method (top row) and for the ANTs_mni method (bottom row). (b) The number of contralesional (left hemisphere) voxels identified by each method. Mean and *SE* are shown over different leave‐one‐out iterations. Note the connectivity in the homologous left fusiform gyrus in the ANTs_cohort template that is not present in the ANTs_mni template

Finally, we tested the effect of normalization methods on functional connectivity in resting state fMRI data in six patients with focal lesions in the territory of the left middle cerebral artery (data described in Section 3.3). Using the right fusiform face area (FFA) as a seed (see Methods), we determined its seed‐to‐voxel connectivity. Significant voxels were identified using both methods at *p* < .01 uncorrected followed by cluster size correction at *p* < .05. We reasoned that more accurate normalization would improve the ability of connectivity analyses to extract the bilateral resting‐state networks that include the FFA. As a result, we compared the number of contralateral suprathreshold voxels identified by ANTs_mni and ANTs_cohort. As shown in Figure 6 and Table 2, ANTs_cohort better identified correlations with the contralateral (left hemisphere) FFA. In contrast, ANTs_mni identified significant voxels located adjacent to the seed region.

**TABLE 2:** Size of significant contralateral connectivity for a right FFA seed

Normalization method	No. of significant contralateral voxels
ANTs_cohort	150.33 (26.58)
ANTs_mni	29.83 (7.51)

*Notes*: In the first row, the functional data were normalized to a cohort‐specific template; in the second row, the functional data were normalized to the MNI‐152 space. Mean and SE are shown over different leave‐one‐out iterations.

## 4 DISCUSSION

The relative accuracy of brain normalization can have a significant impact on reported neuroimaging findings (Avants et al., 2010), particularly in studies of individuals with brain lesions. Here, we demonstrate the advantage of a cohort‐specific template alongside diffeomorphism‐based methods over SPM's unified segmentation method and standard diffeomorphism‐based normalization methods. We show that this approach removes biases in normalization accuracy between individuals with brain lesions and healthy individuals, and that a cohort‐specific template may improve localization of univariate activity and functional connectivity in imaging data with brain lesions.

With respect to the performance of normalization algorithms in imaging data with brain lesions, at least two issues need to be taken into consideration. First, relative to preinjury anatomy, lesions might displace surrounding normal tissue. This difference suggests that evaluation of normalization accuracy for a lesioned brain should consider how the algorithm performs in a reference—that is, representative control brains (Brett et al., 2001). In other words, the accuracy of different registration algorithms in lesioned brains should be assessed in light of their prelesion, ground truth performance. Second, normalization performance should be evaluated using proper accuracy measures that include spatial information such as anatomical landmarks. As an example, naïve registration algorithms that utilize voxel intensity optimization can outperform others despite the profoundly poor mismatch between anatomy in the registered images (Rohlfing, 2012). Thus, evaluating how much landmark displacement occurs after normalization is a more appropriate measure for characterizing registration error, as this metric captures topological displacement and not just voxel intensity mismatches. At this point we acknowledge that the use of manually segmented data for the validation of normalization techniques is limited by the restricted availability of such data. In light of recent studies utilizing large populations with lesions (e.g., the ATLAS stroke database‐Liew et al., 2018), a potential database could be constructed that can serve as a benchmark for future normalization analyses.

In this work, we combine these two ideas by using virtual lesions and the Jaccard overlap between manually segmented regions to evaluate the anatomical displacement caused by each normalization algorithm. Using this methodology, we show that ANTs/diffeomorphism‐based methods are less disrupted by the presence of lesions compared to the widely used SPM unified segmentation method. This finding results from the following factors:

1. The limited number of degrees of freedom that SPM uses. While parsimonious transformations remain an important goal, the quality of the registration (assessed using both local and global measures of anatomical similarity) is positively correlated with the transformation's number of degrees of freedom (Hellier et al., 2003; Klein et al., 2009).
2. The fact that the mapping of SPM can alter the topology of the image in unpredictable fashion, compared to the stable solutions provided by the diffeomorphic transformations. The goal of diffeomorphic‐based image registration is to find a differentiable mapping between images that also has a differentiable inverse. The ANTs toolbox explicitly calculates diffeomorphic transformations and their inverses by optimizing diffeomorphic matching metrics (and appropriate invertibility constraints). The diffeomorphic maps are also symmetric, thus allowing successful registration regardless of which image is considered source and which one is considered target. Together these transformations guarantee that the topology of the images is preserved and that this process is independent of the order in which one inputs the images. This approach stands in contrast to other methods that parameterize the warp differently (e.g., SPM's unified segmentation method that parameterizes the warp based on tissue probability maps).

We used nonparametric tests to find that the ANTs‐based method provided higher normalization accuracy (in terms of difference in medians) compared to the SPM method. However, this method retains an inherent bias because it requires normalization to a template that consists of healthy individuals, thus assuming some form of anatomical correspondence between healthy individuals and patients with brain lesions. However, a patient with a left MCA stroke, for example, might have additional abnormal perilesional tissue in the left hemisphere compared to the MNI‐152 template with which it is matched. To this end, we implemented a new method that involved the creation of a cohort‐specific template consisting of the subjects' images under investigation. This template incorporates the representative anatomy of all the images in the input dataset, rendering it a more anatomically faithful intermediate. Compared to the simple average appearance template from which it starts, the construction process iteratively improves its appearance until convergence. At each step, the use of SyN not only provides a powerful estimate of how similar the template and the images are, but also generates parameters for use in the next step. After a small number of iterations, the process converges to a template in which the individual mappings of the component images achieve maximal accuracy. We found again using nonparametric statistical tests that the template method provided higher median accuracy in both virtual lesion data and real stroke data compared to standard ANTs MNI‐based normalization methods. This result demonstrates that using a common space that better incorporates the morphology of the brains being analyzed can improve normalization results.

An additional issue with directly applying classic ANTs MNI‐based normalization methods to lesioned brains comes when comparing different groups of individuals with brain lesions, or when comparing individuals with brain lesions to healthy individuals. For example, when using the ANTs_mni method, we found a significant relationship between normalization accuracy and lesion size, which is expected given that larger lesions lead to more interpolation due to cost function masking. In addition, we found that normalization accuracy was higher in healthy subjects compared to stroke patients, likely because the MNI‐152 template consists of only healthy subjects, and like‐to‐like (i.e., healthy‐to‐healthy) normalization would be expected to outperform normalization of more dissimilar brains (i.e., lesion‐to‐healthy). Our template method helps to alleviate these issues. First, we found that normalizing to a subject‐specific template lessens the dependence of normalization accuracy on lesion size, as well as the dependence of normalization accuracy on group membership (healthy vs. stroke patients). We argue that the benefits of using a normalization process between brains with similar morphology outweigh the penalty that comes with using cost function masking. Second, we found that when normalizing patients and healthy subjects to the cohort‐specific template, there were no differences in normalization accuracy between groups, likely due to the fact that the template incorporates the morphology of each subject in the study. Importantly, this result held even for stroke patients who were not used to construct the template. Because the template “penalizes” the normalization process equally in both groups, it likely better balances the normalization accuracy between groups. Third, we found that normalization to a cohort‐specific template better co‐registers individuals images with each other, thus allowing improvement of the localization of regional activations at the group level while, at the same time, it improves the identification of significant voxels (i.e., worse normalization methods might lead to increased numbers of false negatives).

### 4.1 Recommendations for template construction

Given the power of the cohort‐specific template method, a consequent question is how to choose which subjects to include in the template in order to adequately represent all the brain data under study. Our suggestion is to construct a template including the images of all subjects (e.g., both patients and healthy controls) under evaluation. The patients can then be registered to the template using SyN with CCFM while the healthy controls are registered using SyN (see Supporting Information S1 for code). This approach will provide a balanced morphology that can enhance normalization accuracy and remove between‐group normalization accuracy differences. However, even in this case topological features of certain substructures in the brain that might be more relevant to a minority of the brains could be lost. For example, mixing a small number of large‐lesion left MCA strokes with multiple right MCA strokes and healthy controls might result in a template that is representative of the majority but fails to capture important left hemispheric anomalies in the left MCA patients. The process of finding the template that adequately represents the majority, without sacrificing the spatial features of the minority of the brains used in each study, remains an active research question and might require the construction and combination of multiple templates.

Another issue to consider when using a cohort‐specific template for group level analysis is the requirement that the data be readily available prior to analysis. Especially in longitudinal studies in which researchers might want to conduct interim analyses, having all the data might not be possible. An encouraging finding in our experiments is that our results could also be derived using an “out‐of‐sample” template representative of stroke patients with similar pathology. This finding speaks to the idea that a template that comes from a different dataset and captures the morphology of the patients under question might be enough to improve accuracy, thus removing the need to have all the study data available. The fact that a template from another stroke dataset was used to improve results in the ATLAS stroke dataset also demonstrates that the utility of the template is not that it captures the relevant MRI, scanner‐specific characteristics of the images that need to be normalized, but rather its ability to represent the structural morphology of the lesion patients. Finally, additional research will be needed to confirm the minimum number of patients required for each template in order to derive optimal normalization accuracy.

One drawback of registering brain images to a template space is interpretability, particularly with respect to previous studies that have reported second‐level results in MNI space. If normalization of results to the MNI‐152 template is necessary, the cohort‐specific template image itself can be normalized to MNI‐152 space, and the resulting transformation can be used to warp individual or group results and labels to MNI‐152 (see Supporting Information S1 for code). Because the two processes of (a) normalizing images to cohort‐specific template space, and (b) normalizing the cohort‐specific template image to MNI‐152, are independent, this method permits results reporting in MNI‐152 space while preserving high normalization accuracy. Researchers might also provide the cohort‐specific template and its transformation to MNI‐152 so that others can interpret and reproduce results in MNI‐space. Steps toward similar efforts have already been taken when imaging templates have been provided for other specific populations (e.g., the use of a pediatric template for imaging studies in children—Avants et al., 2015).

### 4.2 Analysis and reporting guidelines

These results have implications for analyzing and reporting normalization results in future lesion studies of individuals with brain lesions. First, we recommend using open source normalization methods such as ANTs. Some studies use custom‐made scripts (usually combining parts of already established methods) that are often tailored to specific kinds of lesions or imaging data in general. If researchers decide to utilize these custom methods, they should provide their scripts and explain how their methods improve upon state of the art methods. Second, normalization of lesioned brains is an inherently faulty process, as it tries to maximize similarity between brains with a priori different morphologies. This inherent issue suggests that researchers should assess the success of normalization for their specific data set. Assessing landmark displacement after normalization has occurred would be particularly important. Because manual segmentations of regions of interest might not be readily available, an additional step might be required to obtain them. If there is a specific brain structure of interest, then the researcher can prioritize evaluating normalization accuracy on this region. Although in this study we did not systematically evaluate accuracy over different brain regions, it could be the case that some brain regions show worse normalization accuracy than others, thus requiring additional tailoring of the normalization algorithm.

A specific point of concern for previous MRI studies is how normalization accuracy affects second‐level analysis. As our data show, it is important to report normalization accuracy of the groups under question. Of particular concern is the possibility that past reported differences between lesioned patients and controls could be confounded by the normalization method. By evaluating between‐group normalization differences, researchers can be more certain that the reported differences in brain measures between groups are not artificially introduced by the normalization process. Finally, although there is no ground truth with any fMRI result, we showed that based on a priori hypotheses about the location of an fMRI activation/connectivity in the intact cortex of patients, advanced normalization methods can improve localization of these effects. Such results might further validate comparisons between patients and controls, reducing the possibility that suboptimal normalization methods may have introduced artificial differences solely because an effect in healthy individuals cannot be properly localized in patients with lesions.

In conclusion, the importance of lesion studies is undeniable, as they provide critical, causal insights into brain function (Szczepanski & Knight, 2014; Vaidya, Pujara, Petrides, Murray, & Fellows, 2019). Univariate or multivariate analyses have investigated abnormal functional and structural patterns in aphasia (Schwartz et al., 2009; Ivanova et al., 2016), traumatic brain injury (Moreno‐Lopez, Sahakian, Manktelow, Menon, & Stamatakis, 2016; Palacios et al., 2017), and specific cognitive functions in stroke patients (Kayser & D'Esposito, 2013). Despite this wealth of literature, there is still no consensus as to which normalization method should be used, or how comparable results might be when different normalization methods have been used. Hopefully, the current findings provide a foundation for continuing to address these important methodological issues in future patient studies.

## CONFLICT OF INTEREST

The authors declare there are no conflicts of interest.

## DATA AND CODE AVAILABILITY STATEMENT

ANTs, SPM, and CONN are open source packages. Most of the code for processing the data is included in the Supporting Information S1. Scripts for producing the figures were implemented in the Python‐based platform “Jupyter notebook” (https://jupyter.org/) and relied heavily on Seaborn (https://seaborn.pydata.org/). The Jupyter notebook and its accompanying data will be uploaded after publication to https://github.com/ioannispappas322. The LPAB40 and ATLAS imaging data are publicly available. Virtual lesion data can be constructed using the provided scripts or requested from the corresponding author (IP). Imaging data for the rest of the patients and healthy controls can be requested via communication to the corresponding author (IP).

## Supporting information

**Appendix S1**. Supporting Information.
