# Supplementary material for: Improved normalization of lesioned brains via cohort‐specific templates
Source: Hum Brain Mapp. 2021 Jun 18;42(13):4187–204. doi: 10.1002/hbm.25474 (PMC8356997; doi:10.1002/hbm.25474)
Supplement: Supplementary file 1 — Appendix S1. Supporting Information. [file HBM-42-4187-s001.docx]

**Supplementary Material**

A. Supplementary Figures


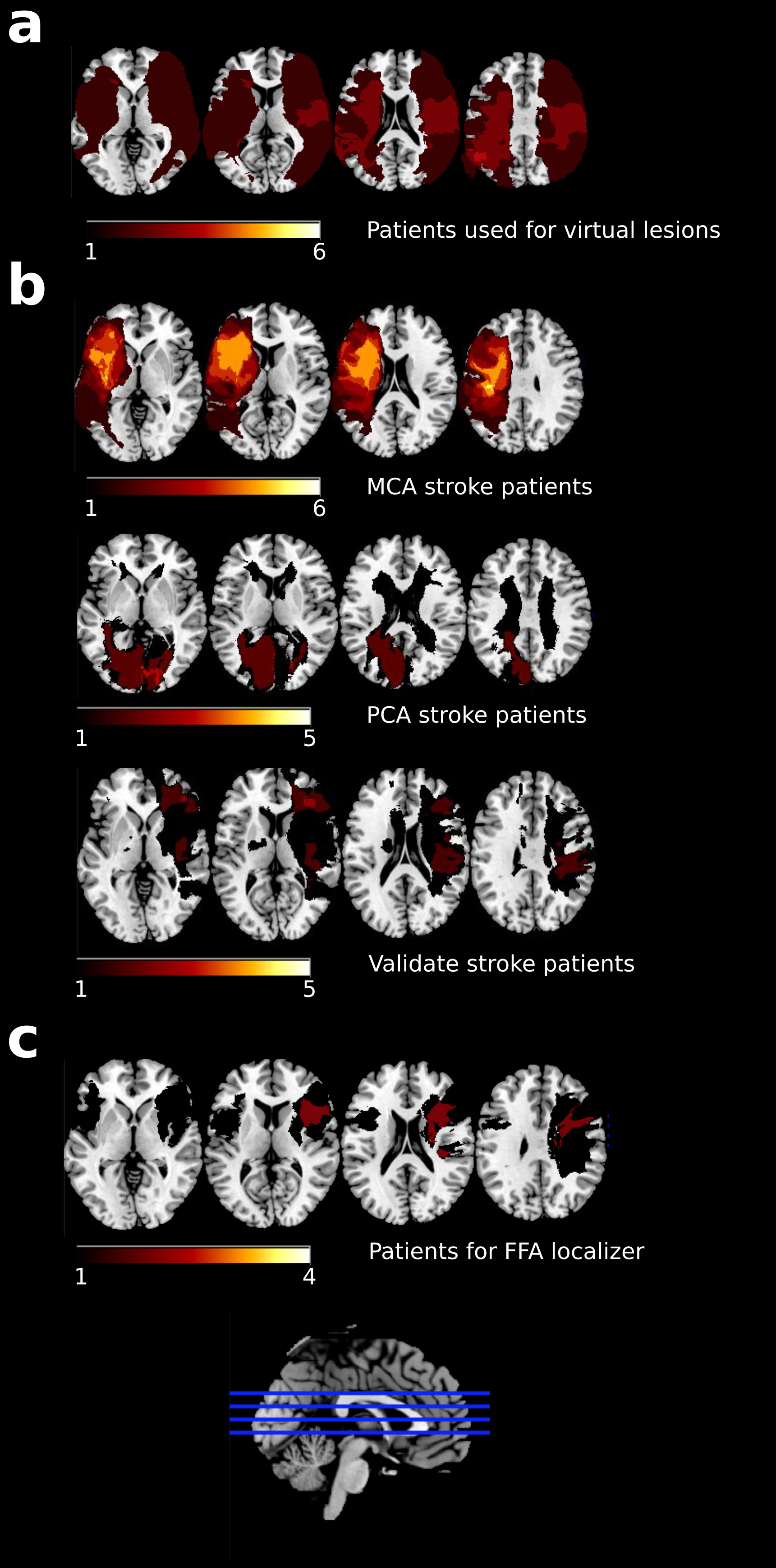


**Supplementary Figure1-Lesion distribution for patients used in experiments.** a) Heatmap showing the spatial distribution for the lesions that were used in the creation of the virtual lesioned brains (experiment 1). These lesions were derived from structural imaging data of stroke patients. b) In experiment 3 we used three types of stroke patients with different lesion locations: left middle cerebral artery (MCA stroke patients-those were also used in Experiment 5), posterior cerebral artery (PCA stroke patients), and right MCA for validation (validate stroke patients). Heatmaps represent their respective spatial distributions. c) Heatmap representing the spatial distribution of the lesions in subjects with frontal lesions who were used in the face localizer experiment (experiment 4).


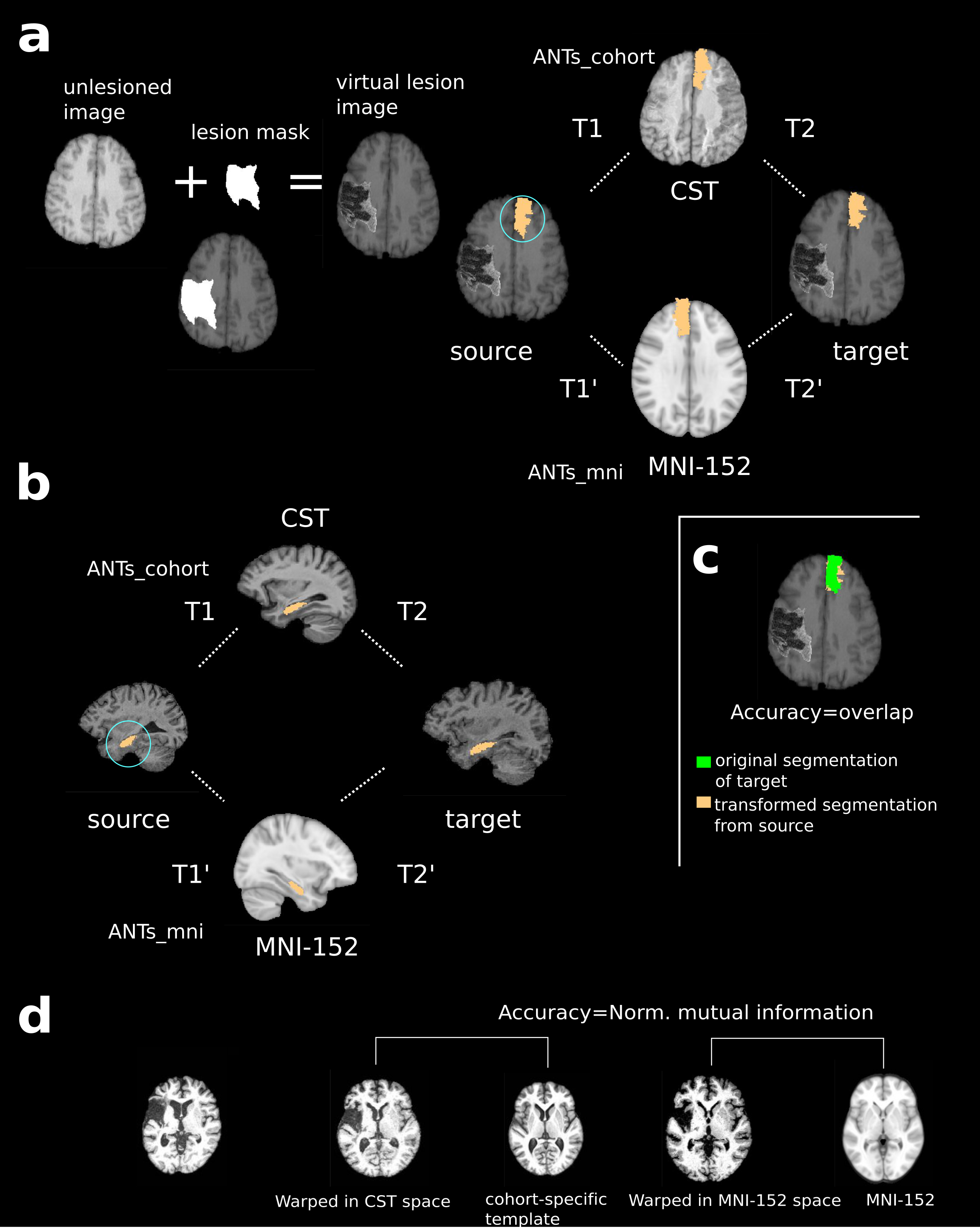


**Supplementary Figure 2-Overview of normalization accuracy evaluation for experiments 1,2,3.** a) For experiment 1, we used the LPAB40 data that had manual segmentations of regions of interest (ROIs). Using these data and lesion segmentations from an independent stoke patient cohort, we created virtual lesion images by inserting lesions into the unlesioned LPAB40 data. For each registration algorithm, its accuracy was assessed for pairs of images (source, target) as follows. Source and target were non-linearly registered to a common space (either MNI-152 space for ANTs_mni and SPM unified segmentation/SPM_us, or a cohort-specific template/CST space in the case of ANTs_cohort). ROI labels of the source image were then warped to the common space and inversely warped to the target’s space. b) For experiment 2, we used the segmented labels of the bilateral hippocampus to obtain warped images to the target’s space in identical fashion to experiment 1. c) Accuracy was then quantified by the overlap between the warped images of the source and the manually segmented labels of the target. In the case of virtual lesions we calculated the absolute difference in accuracy, defined as the difference between the accuracy obtained using the virtual lesions and the accuracy obtained using the healthy images from which the virtual lesions were derived. d) In experiment 3, due to a lack of manual segmentations we assessed accuracy by calculating an information-theoretic measure (normalized mutual information-NMI) between the lesioned brain and the MNI-152 template in the case of ANTs_mni, and cohort-specific template space in the case of the ANTs_cohort method.


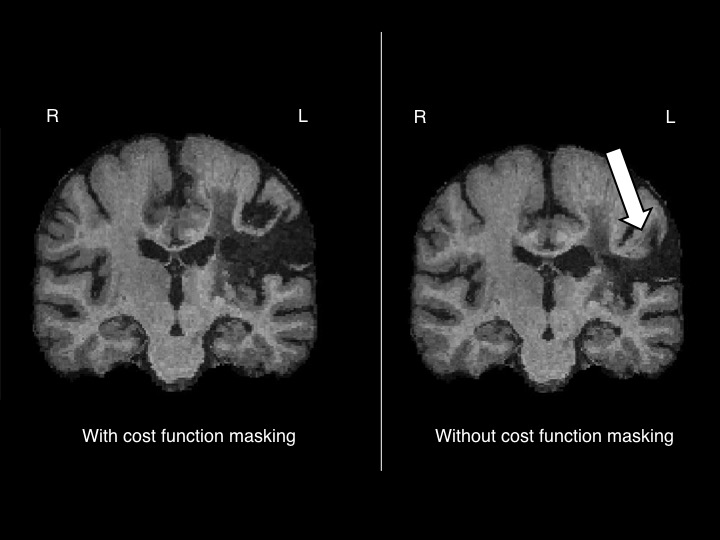


**Supplementary Figure 3-The effect of cost function masking on ANTs normalization.** SyN without cost function masking performs poorly, as the perilesional space appears to be affected by the normalization process. To showcase this point we show one particular lesion dataset in which we used ANTs_mni normalization results with and without cost function masking. One can see that the perilesional space is “drawn” towards the lesion site. We thus strongly recommend the use of SyN cost function masking as the default method.


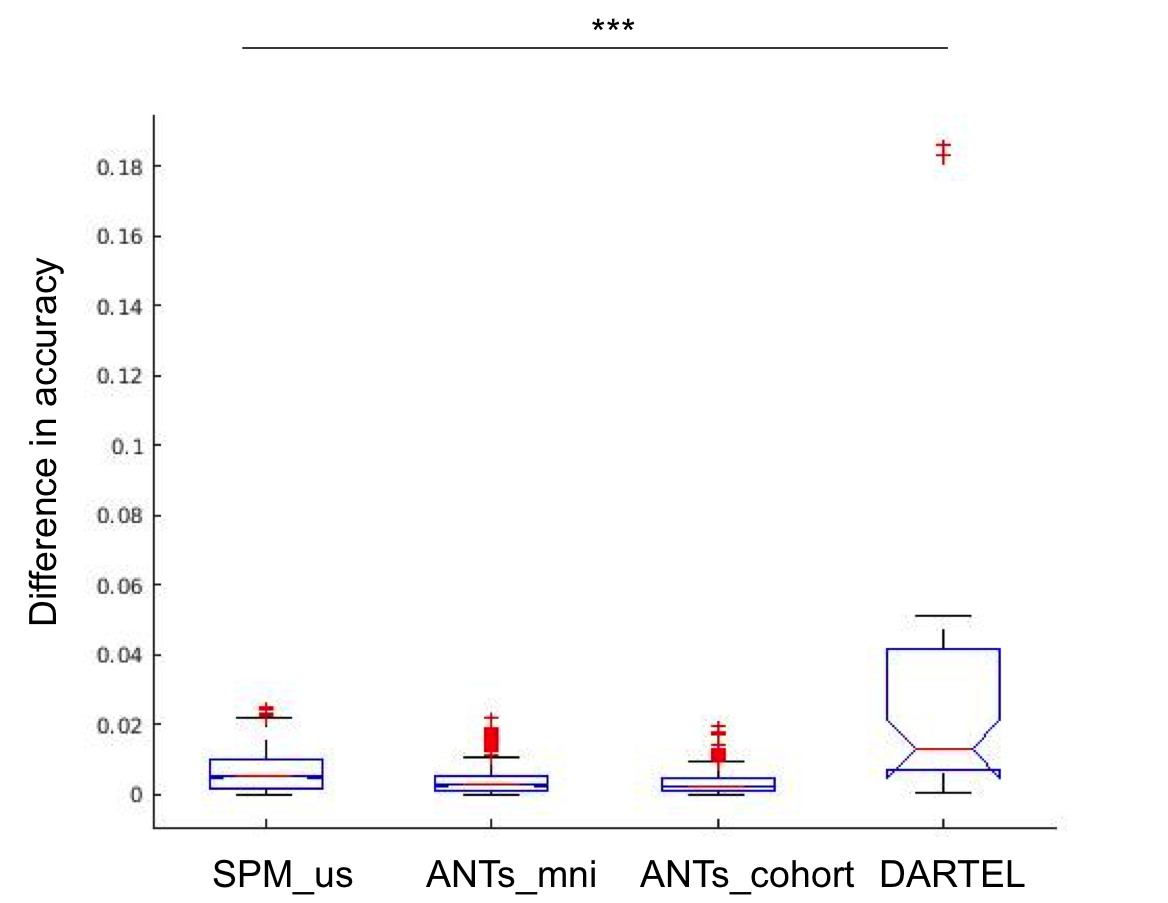


**Supplementary Figure 4-Comparison of the SPM_us and ANTs methods to DARTEL.** An alternative method to SPM’s unified segmentation method for normalizing images is DARTEL (Diffeomorphic Anatomical Registration Through Exponential Lie Algebra). DARTEL’s normalization involves iteratively computing a template of the gray matter and white matter tissue probability maps from all subjects and registering all subjects' tissue probability maps to the template. DARTEL's registrations are parameterized by a single diffeomorphic field compared to the bi-directional diffeomorphism of ANTs. As part of the default implementation, DARTEL operates on whole-brain images. It also provides the resulting deformations used for optimally matching the images and the template. We used default parameters for the DARTEL template construction (6 outer iterations with smoothing=16, 8, 4, 2, 1, 0.5 respectively, regularization=0.01 for stability of the optimization). Code is available in the next section. We used DARTEL on the virtual lesion data of experiment 1**.** DARTEL underperformed compared to the other methods (Kruskal-Wallis ANOVA chi-squared=164.12, P<0.001, post hoc test Bonferroni corrected P<0.001 for all comparisons of DARTEL to the other methods). Due to this result we decided not to explore DARTEL further, although future work might assess its utility by searching the parameter space for parameters (e.g. amount of smoothing and warping regularization) suitable for lesion studies. The y-axis presents the absolute difference between normalization accuracy of virtual lesion images and their corresponding unlesioned images. Whiskers represent the 1.5 inter-quartile range (IQR). Horizontal lines inside the boxplots represent median values. *** P<0.001 Kruskal-Wallis ANOVA.

B. Supplementary code

#for all the ANTs calls make sure ANTs is in your path

#e.g. in your bash_profile add these two lines

#ANTSPATH=/home/Software/ants-2.2.0/bin/

#export PATH=${ANTSPATH}

B1. Creating virtual lesions

#!/bin/bash

# Input:

# your healthy subjects are im1, im2 with t1 filenames t1mprage_$sub_hc.nii.gz

# your lesion subjects are pat1 pat2 with t1 filenames t1mprage_$sub.nii.gz

# your lesion masks for the lesion subjects with filenames sub_$sub_mask.nii.gz

# Output: virtual lesion brains

#

# Required toolboxes: fsl (install following instructions provided here #https://fsl.fmrib.ox.ac.uk/fsl/fslwiki/FslInstallation), and ANTs (install following #instructions provided here: #http://stnava.github.io/ANTs/-make sure both of them are #in your path)

for source in (pat1,pat2)

do

for target in (im1,im2)

do

name=virt3_${source}

# prepare a directory to store the virtual lesion image

mkdir /path/to/healthy/${target}/forks/${name}

# if there is no brain for each one of the patients you can run

# antsBrainExtraction.sh -d 3 -a #/path/to/stroke/${source}/anat/t1mprage_${source}.nii.gz -e #/path/to/priorsants/T_template_prior.nii.gz -m #/path/to/priorsants/T_template_prior_BrainCerebellumMask.nii.gz -o #/path/to/stroke/${source}/ANTs_results/Output

# the mask also needs to be bin inversed

# e.g. fslmaths /path/to/stroke/${source}/mask/sub_${source}_mask.nii.gz -binv sub_${source}_non_lesion.nii.gz

#start the process

fslmaths /path/to/stroke/${source}/anat/t1mprage_${source}.nii.gz -mul /path/to/stroke/${source}/mask/sub_${source}_mask.nii.gz /path/to/healthy/${target}/anat/patch.nii.gz

flirt -in /path/to/healthy/${target}/anat/t1mprage_${target}_hc.nii.gz -ref /path/to/stroke/${source}/anat/t1mprage_${source}.nii.gz -out /path/to/healthy/${target}/anat/resliced_${source}_${target}.nii.gz

# you will also need a brain extraction of this resliced healthy target

# e.g. somewhere like /path/to/healthy/${target}/forks/${name}/ #/OutputBrainExtractionBrain.nii.gz

# Brett factors:

fslmaths /path/to/stroke/${source}/ANTs_results/OutputBrainExtractionBrain.nii.gz -mul /path/to/stroke/${source}/mask/sub_${source}_non_lesion.nii.gz /path/to/stroke/${source}/cut_image

var4="$(fslstats /path/to/stroke/${source}/cut_image.nii.gz -M)"

var5="$(fslstats /path/to/healthy/${target}/forks/${name} /OutputBrainExtractionBrain.nii.gz -M)"

fslmaths /path/to/healthy/${target}/anat/resliced_${source}_${target}.nii.gz -mul /path/to/stroke/${source}/mask/sub_${source}_non_lesion.nii.gz /path/to/healthy/${target}/anat/patch2.nii.gz

fslmaths /path/to/healthy/${target}/anat/patch.nii.gz -mul ${var5} -div ${var4} /path/to/healthy/${target}/anat/patch_r2.nii.gz

# finalize

fslmaths /path/to/healthy/${target}/anat/patch2.nii.gz -add /path/to/healthy/${target}/anat/patch_r2.nii.gz /path/to/healthy/${target}/forks/${name}/${name}_s.nii.gz

# copy and rename the mask

cp /path/to/stroke/${source}/mask/sub_${source}_mask.nii.gz /path/to/healthy/${target}/forks/${name}

mv /path/to/healthy/${target}/forks/${name}/sub_${source}_mask.nii.gz /path/to/healthy/${target}/forks/${name}/sub_${target}_mask_${name}.nii.gz

done

done

B2. Creating a template

#!/bin/bash

# ANTs toolbox needs to be in the path

cd /path/of/analysis

# 1. create the template from anatomical t1s; type buildtemplateparallel.sh to change #options that match your study

buildtemplateparallel.sh -d 3 -o output /path/to/t1s/*.nii.gz

# 2. brain extraction of the template-this step requires priors (provided freely with ANTs)

antsBrainExtraction.sh -d 3 -a outputtemplate.nii.gz -e /path/to/priorsants/T_template_prior.nii.gz -m /path/to/priorsants/T_template_prior_BrainCerebellumMask.nii.gz -o output

# 3. cortical thickness command for obtaining tissue segmentations for the template-this step requires priors (provided freely with ANTs)

antsCorticalThickness.sh -d 3 -a outputtemplate.nii.gz -e /path/to/priorants/T_template_prior.nii.gz -m /path/to/priorsants/T_template_prior_BrainCerebellumMask.nii.gz -p /path/to/priorsants/tissue_priors%d.nii.gz -o seg2

B3. Normalization using ANTs (ANTs_mni, ANTs_cohort)

#!/bin/bash

# for ANTs_mni

dd=1

# for ANTs_cohort

dd=2

# data

if [ ${dd} = 1 ]

then

# MNI_template

TEMPLATE_NAME=/path/to/MNI/MNI152_T1_1mm.nii.gz

# extra priors for segmentation

PRIOR_TEMPLATE=/path/to/MNI/MNI152_T1_1mm.nii.gz

PRIOR_TEMPLATE_MASK=/path/to/MNI/MNI152_T1_1mm_BrainCerebellumMask.nii.gz

PRIOR_BRAIN=/path/to/MNI/MNI152_T1_1mm_BrainExtraction.nii.gz

PRIOR_TEMPLATE_TISSUE=/path/to/MNI/BrainSegmentation%d.nii.gz

elif [ ${dd} = 2 ]

then

# CST

TEMPLATE_NAME=/path/to/template/outputtemplate.nii.gz

# extra priors for segmentation (obtained as in B2)

PRIOR_TEMPLATE=/path/to/template/outputtemplate.nii.gz

PRIOR_TEMPLATE_MASK=/path/to/template/BrainCerebellumMask.nii.gz

PRIOR_BRAIN=/path/to/template/BrainExtraction.nii.gz

PRIOR_TEMPLATE_TISSUE=/path/to/template/priors/BrainSegmentation%d.nii.gz

fi

# for one subject example

subj=111

# 1. paths setup

DATA_IOANNIS=/path/to/data

forks_folder=ANTs_analysis

FORKS_PATH=${DATA_IOANNIS}/${subj}/forks/${forks_folder}

mkdir ${DATA_IOANNIS}/${subj}/forks/${forks_folder}

cd ${DATA_IOANNIS}/${subj}/forks/${forks_folder}

# structural

structural_path=${DATA_IOANNIS}/${subj}/t1mprage.nii.gz

# mask path (required for CCFM)

lesion_mask_path=${DATA_IOANNIS}/${subj}/mask.nii.gz

# 2. bin inverse the mask (for healthy control this is not needed)

fslmaths ${lesion_mask_path} -binv ${DATA_IOANNIS}/${subj}/mask_non_lesion.nii.gz

# 3. brain extraction using template (optional-you might just want to normalize the t1 directly)

antsBrainExtraction.sh -d 3 -o Output -a ${structural_path} -e ${PRIOR_TEMPLATE} -m ${PRIOR_TEMPLATE_MASK}

# 4. segmentation (optional)

antsCorticalThickness.sh -d 3 -a ${structural_path} -e ${PRIOR_TEMPLATE} -m ${PRIOR_TEMPLATE_MASK} -p ${PRIOR_TEMPLATE_TISSUE} -o seg2

# 5. registration to MNI template with CCFM (for healthy controls –x is obsolete)

# here you can do brain to brain…

#antsRegistrationSyN.sh -d 3 -f OutputBrainExtractionBrain.nii.gz -m ${PRIOR_BRAIN} -x ${DATA_IOANNIS}/${subj}/mask_non_lesion.nii.gz -o ATR

# …or full t1 to full t1.

antsRegistrationSyN.sh -d 3 -f ${structural_path} -m ${TEMPLATE_NAME} -x ${DATA_IOANNIS}/${subj}/mask_non_lesion.nii.gz -o ATR

#6. apply inverse transformations to bring everything to the template space

antsApplyTransforms -d 3 -i ${structural_path} -o t1mprage_MNI.nii.gz -r ${TEMPLATE_NAME} -t [ATR0GenericAffine.mat,1] -t ATR1InverseWarp.nii.gz -n Linear --float 1

antsApplyTransforms -d 3 -i OutputBrainExtractionBrain.nii.gz -o OutputBrainExtractionBrain_MNI.nii.gz -r ${TEMPLATE_NAME} -t [ATR0GenericAffine.mat,1] -t ATR1InverseWarp.nii.gz -n Linear --float 1

antsApplyTransforms -d 3 -i ${lesion_mask_path} -o mask_MNI.nii.gz -r ${TEMPLATE_NAME} -t [ATR0GenericAffine.mat,1] -t ATR1InverseWarp.nii.gz -n NearestNeighbor --float 1

antsApplyTransforms -d 3 -i seg2BrainSegmentationPosteriors1.nii.gz -o seg2BrainSegmentationPosteriors1_MNI.nii.gz -r ${TEMPLATE_NAME} -t [ATR0GenericAffine.mat,1] -t ATR1InverseWarp.nii.gz -n NearestNeighbor --float 1

antsApplyTransforms -d 3 -i seg2BrainSegmentationPosteriors2.nii.gz -o seg2BrainSegmentationPosteriors2_MNI.nii.gz -r ${TEMPLATE_NAME} -t [ATR0GenericAffine.mat,1] -t ATR1InverseWarp.nii.gz -n NearestNeighbor --float 1

antsApplyTransforms -d 3 -i seg2BrainSegmentationPosteriors3.nii.gz -o seg2BrainSegmentationPosteriors3_MNI.nii.gz -r ${TEMPLATE_NAME} -t [ATR0GenericAffine.mat,1] -t ATR1InverseWarp.nii.gz -n NearestNeighbor --float 1

B4. SPM normalization

%required: SPM e.g. addpath /path/to/spm in your MATLAB console

%% SPM normalization

Defaults = spm_get_defaults;

spm_jobman('initcfg')

% Normalize

matlabbatch{1}.spm.spatial.normalise.estwrite.subj.vol = {anat.nii};

matlabbatch{1}.spm.spatial.normalise.estwrite.subj.resample = {anat.nii};

matlabbatch{1}.spm.spatial.normalise.estwrite.eoptions.biasreg = 0.0001;

matlabbatch{1}.spm.spatial.normalise.estwrite.eoptions.biasfwhm = 60;

matlabbatch{1}.spm.spatial.normalise.estwrite.eoptions.tpm = {'/path/to/spm12/tpm/TPM.nii'};

matlabbatch{1}.spm.spatial.normalise.estwrite.eoptions.affreg = 'mni';

matlabbatch{1}.spm.spatial.normalise.estwrite.eoptions.reg = [0 0.001 0.5 0.05 0.2];

matlabbatch{1}.spm.spatial.normalise.estwrite.eoptions.fwhm = 0;

matlabbatch{1}.spm.spatial.normalise.estwrite.woptions.warp.write = [1 1];

matlabbatch{1}.spm.spatial.normalise.estwrite.eoptions.samp = 3;

matlabbatch{1}.spm.spatial.normalise.estwrite.woptions.bb = [-78 -112 -70

78 76 85];

matlabbatch{1}.spm.spatial.normalise.estwrite.woptions.vox = [2 2 2];

matlabbatch{1}.spm.spatial.normalise.estwrite.woptions.interp = 4;

matlabbatch{1}.spm.spatial.normalise.estwrite.woptions.prefix = 'w';

spm_jobman('run',matlabbatch)

clear matlabbatch

%inverse normalize (for example in the case of the ATLAS hippocampal segmentations )

%this requires the inverse deformation field produced by the unified segmentation method %

matlabbatch{1}.spm.spatial.normalise.write.subj.def = {iy_inverse_field.nii};

matlabbatch{1}.spm.spatial.normalise.write.subj.resample = {hippocampus_MNI.nii};

matlabbatch{1}.spm.spatial.normalise.write.woptions.bb = [-78 -112 -70

78 76 85];

matlabbatch{1}.spm.spatial.normalise.write.woptions.vox = [1 1 1];

matlabbatch{1}.spm.spatial.normalise.write.woptions.interp = 0;

matlabbatch{1}.spm.spatial.normalise.write.woptions.prefix = 'iw';

spm_jobman('run',matlabbatch)

clear matlabbatch

B5. DARTEL

This script produces a DARTEL template from two t1-weighted images and the accompanying deformation fields that can take images from native space to template space.

matlabbatch{1}.spm.tools.dartel.warp.images = {{‘t1.nii’,’t2.nii’}};

matlabbatch{1}.spm.tools.dartel.warp.settings.template = 'Template';

matlabbatch{1}.spm.tools.dartel.warp.settings.rform = 0;

matlabbatch{1}.spm.tools.dartel.warp.settings.param(1).its = 3;

matlabbatch{1}.spm.tools.dartel.warp.settings.param(1).rparam = [4 2 1e-06];

matlabbatch{1}.spm.tools.dartel.warp.settings.param(1).K = 0;

matlabbatch{1}.spm.tools.dartel.warp.settings.param(1).slam = 16;

matlabbatch{1}.spm.tools.dartel.warp.settings.param(2).its = 3;

matlabbatch{1}.spm.tools.dartel.warp.settings.param(2).rparam = [2 1 1e-06];

matlabbatch{1}.spm.tools.dartel.warp.settings.param(2).K = 0;

matlabbatch{1}.spm.tools.dartel.warp.settings.param(2).slam = 8;

matlabbatch{1}.spm.tools.dartel.warp.settings.param(3).its = 3;

matlabbatch{1}.spm.tools.dartel.warp.settings.param(3).rparam = [1 0.5 1e-06];

matlabbatch{1}.spm.tools.dartel.warp.settings.param(3).K = 1;

matlabbatch{1}.spm.tools.dartel.warp.settings.param(3).slam = 4;

matlabbatch{1}.spm.tools.dartel.warp.settings.param(4).its = 3;

matlabbatch{1}.spm.tools.dartel.warp.settings.param(4).rparam = [0.5 0.25 1e-06];

matlabbatch{1}.spm.tools.dartel.warp.settings.param(4).K = 2;

matlabbatch{1}.spm.tools.dartel.warp.settings.param(4).slam = 2;

matlabbatch{1}.spm.tools.dartel.warp.settings.param(5).its = 3;

matlabbatch{1}.spm.tools.dartel.warp.settings.param(5).rparam = [0.25 0.125 1e-06];

matlabbatch{1}.spm.tools.dartel.warp.settings.param(5).K = 4;

matlabbatch{1}.spm.tools.dartel.warp.settings.param(5).slam = 1;

matlabbatch{1}.spm.tools.dartel.warp.settings.param(6).its = 3;

matlabbatch{1}.spm.tools.dartel.warp.settings.param(6).rparam = [0.25 0.125 1e-06];

matlabbatch{1}.spm.tools.dartel.warp.settings.param(6).K = 6;

matlabbatch{1}.spm.tools.dartel.warp.settings.param(6).slam = 0.5;

matlabbatch{1}.spm.tools.dartel.warp.settings.optim.lmreg = 0.01;

matlabbatch{1}.spm.tools.dartel.warp.settings.optim.cyc = 3;

matlabbatch{1}.spm.tools.dartel.warp.settings.optim.its = 3;

Then given the deformation fields one can transform images to template space

matlabbatch{1}.spm.tools.dartel.crt_warped.flowfields = {t1};

matlabbatch{1}.spm.tools.dartel.crt_warped.images = {{label1}};

matlabbatch{1}.spm.tools.dartel.crt_warped.jactransf = 0;

matlabbatch{1}.spm.tools.dartel.crt_warped.K = 6;

matlabbatch{1}.spm.tools.dartel.crt_warped.interp = 0;

Or given the inverse deformation fields one can transform images from template space to native space

matlabbatch{1}.spm.tools.dartel.crt_iwarped.flowfields = {t2};

matlabbatch{1}.spm.tools.dartel.crt_iwarped.images = {label2};

matlabbatch{1}.spm.tools.dartel.crt_iwarped.K = 6;

matlabbatch{1}.spm.tools.dartel.crt_iwarped.interp = 0;

matlabbatch{1}.spm.tools.dartel.crt_iwarped.vox = [1 1 1];

B6. Evaluation of normalization (Jaccard, NMI)

# required: Python 3- obtained via Anaconda #https://www.anaconda.com/products/individual

from __future__ import print_function

from __future__ import division

# - import common modules

import numpy as np

import matplotlib.pyplot as plt

import nibabel as nib

from subprocess import call

from scipy.special import gamma,psi

from scipy import ndimage

from scipy.linalg import det

from numpy import pi

import scipy

import shlex

import subprocess

import nibabel as nib

import sys

import pdb

EPS = np.finfo(float).eps

from nipype.algorithms.metrics import Overlap

def overlap_(x, y):

overlap = Overlap()

overlap.inputs.volume1 = x

overlap.inputs.volume2 = y

res = overlap.run()

out = res.outputs

return out.jaccard

def mutual_information_2d(x, y, sigma=1, normalized=True):

"""

Computes (normalized) mutual information between two 1D variate from a

joint histogram.

Parameters

----------

x : 1D array

first variable

y : 1D array

second variable

sigma: float

sigma for Gaussian smoothing of the joint histogram

Returns

-------

nmi: float

the computed similariy measure

"""

bins = (256, 256)

jh = np.histogram2d(x, y, bins=bins)[0]

# smooth the jh with a gaussian filter of given sigma

ndimage.gaussian_filter(jh, sigma=sigma, mode='constant',

output=jh)

# compute marginal histograms

jh = jh + EPS

sh = np.sum(jh)

jh = jh / sh

s1 = np.sum(jh, axis=0).reshape((-1, jh.shape[0]))

s2 = np.sum(jh, axis=1).reshape((jh.shape[1], -1))

# Normalised Mutual Information of:

# Studholme, jhill & jhawkes (1998).

# "A normalized entropy measure of 3-D medical image alignment".

# in Proc. Medical Imaging 1998, vol. 3338, San Diego, CA, pp. 132-143.

if normalized:

mi = ((np.sum(s1 * np.log(s1)) + np.sum(s2 * np.log(s2)))

/np.sum(jh * np.log(jh))) - 1

else:

mi = ( np.sum(jh * np.log(jh)) - np.sum(s1 * np.log(s1))

- np.sum(s2 * np.log(s2)))

return mi

if __name__=='__main__':

im1 = '/path/to/im1.nii.gz'

im1_nb = nib.load(im1)

im1_d = im1_nb.get_data()

im2 = '/path/to/im2.nii.gz'

im2_nb = nib.load(im2)

im2_d = im2_nb.get_data()

sh1 = im1_d.shape

sh1 = im2_d.shape

t1 = im1_d.reshape((sh1[0]*sh1[1])*sh1[2])

t2 = im2_d.reshape((sh2[0]*sh2[1])*sh2[2])

# Jaccard

J = overlap_(im1,im2)

# NMI

NMI= mutual_information_2d(t1,t2)

B6. Registering a cohort specific template and any maps that come with it (e.g. second level analysis maps) to MNI space

#!/bin/bash

#use SyN to register the template to MNI

antsRegistrationSyN.sh -d 3 -f ${MNI_TEMPLATE} -m ${TEMPLATE_NAME} -o ATR

# use the resulting transformation to map all the cohort-specific template results to MNI #space

antsApplyTransforms -d 3 -i ${MYMAP}.nii.gz -o ${MYMAP_INMNI} -r ${MNI_TEMPLATE} -t [ATR0GenericAffine.mat] -t ATR1Warp.nii.gz -n Linear --float 1
